# Functional connectome fingerprinting across the lifespan

**DOI:** 10.1162/netn_a_00320

**Published:** 2023-10-01

**Authors:** Frédéric St-Onge, Mohammadali Javanray, Alexa Pichet Binette, Cherie Strikwerda-Brown, Jordana Remz, R. Nathan Spreng, Golia Shafiei, Bratislav Misic, Étienne Vachon-Presseau, Sylvia Villeneuve

**Affiliations:** Integrated Program in Neuroscience, Faculty of Medicine, McGill University, Montreal, Canada; Research Center of the Douglas Mental Health University Institute, Montreal, Canada; Clinical Memory Research Unit, Faculty of Medicine, Lund University, Lund, Sweden; Department of Neurology and Neurosurgery, Montreal Neurological Institute, McGill University, Montreal, Canada; McConnell Brain Imaging Centre, Montreal Neurological Institute, McGill University, Montreal, Canada; Faculty of Dental Medicine and Oral Health Sciences, McGill University, Montreal, Canada; Department of Anesthesia, Faculty of Medicine, McGill University, Montreal, Canada; Alan Edwards Centre for Research on Pain (AECRP), McGill University, Montreal, Canada

**Keywords:** Functional connectome fingerprinting, fMRI, Lifespan

## Abstract

Systematic changes have been observed in the functional architecture of the human brain with advancing age. However, functional connectivity (FC) is also a powerful feature to detect unique “connectome fingerprints,” allowing identification of individuals among their peers. Although fingerprinting has been robustly observed in samples of young adults, the reliability of this approach has not been demonstrated across the lifespan. We applied the fingerprinting framework to the Cambridge Centre for Ageing and Neuroscience cohort (*n* = 483 aged 18 to 89 years). We found that individuals are “fingerprintable” (i.e., identifiable) across independent functional MRI scans throughout the lifespan. We observed a U-shape distribution in the strength of “self-identifiability” (within-individual correlation across modalities), and “others-identifiability” (between-individual correlation across modalities), with a decrease from early adulthood into middle age, before improving in older age. FC edges contributing to self-identifiability were not restricted to specific brain networks and were different between individuals across the lifespan sample. Self-identifiability was additionally associated with regional brain volume. These findings indicate that individual participant-level identification is preserved across the lifespan despite the fact that its components are changing nonlinearly.

## Author Summary

Important interindividual differences are increasingly identified in functional connectivity studies. However, most of the work to date has focused on samples of younger adults, ignoring older individuals in which brains undergo significant transformation. Here, we examine individual-level patterns of functional connectivity—that is, connectome fingerprints—across the adult lifespan. Our results suggest that connectome fingerprints can reliably predict individuals from independent brain scans across the lifespan. Additionally, regions contributing to fingerprints differed from person to person, emphasizing the extent of interindividual differences in the brain. Specific patterns of brain volume, associated with normative age-related atrophy, may impact the identification of individual-specific functional connectivity patterns. Our findings highlight the importance of interindividual variability in functional connectivity, regardless of participant age, when assessing individual differences in brain function.

## INTRODUCTION

The structural and functional organization of the brain is reliably consistent within species because of strong genetic control of this biological system (Gómez-Robles et al., 2015). In humans, however, substantial intraindividual variability has been found at a fine-grained level (Amico & Goñi, 2018; Finn et al., 2015; Mansour et al., 2021; Mueller et al., 2013). An emerging body of evidence suggests that interindividual differences in brain connectomes are sufficient to match brain scans and effectively identify individuals among large datasets of brain images. These “signatures” or “fingerprints” (Finn et al., 2015, 2017) are stable over years (Guo et al., 2012; Horien et al., 2019; Jalbrzikowski et al., 2020; Ousdal et al., 2020) and between scan conditions (Finn et al., 2015, 2017; Vanderwal et al., 2017), and they are found using other brain scanning modalities such as magnetoencephalography (da Silva Castanheira et al., 2021). Individual participant identifiability is observable in homogeneous samples of young adults (Finn et al., 2015; Mueller et al., 2013), yet older adults have been relatively neglected in the literature.

Cross-sectional studies comparing older and younger adult groups have revealed substantial differences in functional connectivity (FC) (Chen et al., 2016; Setton et al., 2023; Zonneveld et al., 2019), raising questions about the reliability of FC fingerprinting in older adults. Our aims were to (a) test the stability of fingerprint identification accuracy (i.e., uniqueness of the connectomes, which facilitates individual participant identification; Finn et al., 2015), (b) determine self-identifiability (i.e., a continuous variable measuring within-individual similarity across independent observations; Amico & Goñi, 2018), and (c) characterize others-identifiability (i.e., a continuous variable measuring how similar an individual is relative to others; Amico & Goñi, 2018). These aims were examined across the lifespan, spanning the full connectome, as well as within and between large-scale networks, using functional magnetic resonance imaging (fMRI). We then determined which functional connections between regions (i.e., edges) reliably contributed to identification and how these patterns varied across the lifespan. Finally, we explored the association between identifiability and brain volume, a significant predictor of participant age (Gonneaud et al., 2021). We derived fingerprint metrics using a pair of fMRI conditions (resting state and sensorimotor task) from cognitively healthy adults across the lifespan in the Cambridge Centre for Ageing and Neuroscience (Cam-CAN) cohort (*n* = 483; ages 18–89 years) (Shafto et al., 2014; Taylor et al., 2017). Our results indicate that fingerprint identifiability is a reliable metric across the lifespan. We also show that self- and others-identifiability measures had nonlinear distributions across the lifespan. Self- and others-identifiability was high in young adults, decreased into middle age, then increased again into older adulthood. Elastic net models revealed that the fingerprinting methodology identifies dominant individual-specific features of FC, reliably demarcating unique patterns for each healthy adult at each decade of life. Finally, self-identifiability, but not others-identifiability, was associated with brain volume in regions known to atrophy in the context of normative aging. Overall, the results suggest that intraindividual variability in the organization of the human brain, particularly in older adults, warrants consideration in parallel with normative trajectories of age-related brain change.

## RESULTS

Analyses were performed on 483 individuals of the Cam-CAN cohort (Shafto et al., 2014; Taylor et al., 2017) aged 18 to 89 years. Participants were included if they had at least two fMRI scans (rest and sensorimotor task modalities) passing quality control. Demographic information is presented in Table 1. About half of our sample was composed of females. Most participants were right-handed. The final sample comprised at least 50 individuals in each decade of life, except individuals between 80 and 89 years of age, with only 34 included participants.

**Table 1.** Demographics information. Handedness was measured as a continuous variable from −100 (fully left-handed) to 100 (full right-handed). Motion is reported as the average frame displacement for each modality. The number of frames is the number of fMRI frames remaining after removing frames with excessive motion. Counts are presented for categorical data, while average and standard deviation are presented for continuous data.

**Decades of age variables**	**18–29 (*n* = 71)**	**30–39 (*n* = 88)**	**40–49 (*n* = 95)**	**50–59 (*n* = 68)**	**60–69 (*n* = 71)**	**70–79 (*n* = 56)**	**80–89 (*n* = 34)**	**Overall (*n* = 483)**
**Sex (F)**	40	43	50	34	30	27	15	239
**Sex (M)**	31	45	45	34	41	29	19	244
	**Mean (*SD*)**							
**Age**	25.00 (3.50)	34.83 (2.64)	44.54 (3.06)	54.51 (2.89)	64.45 (2.84)	75.16 (2.97)	83.06 (2.39)	50.49 (18.24)
**Handedness**	75.01 (49.57)	78.08 (48.75)	74.22 (55.35)	75.91 (54.06)	77.15 (52.92)	85.09 (42.77)	86.53 (36.06)	77.82 (50.12)
**Motion (mm)_(Rest)_**	0.175 (0.048)	0.173 (0.047)	0.196 (0.057)	0.203 (0.050)	0.221 (0.041)	0.235 (0.047)	0.238 (0.046)	0.201 (0.054)
**Motion (mm)_(Task)_**	0.153 (0.044)	0.147 (0.042)	0.170 (0.051)	0.182 (0.049)	0.198 (0.046)	0.209 (0.047)	0.230 (0.052)	0.178 (0.053)
**Number of frames_(Rest)_**	238.06 (31.14)	246.08 (26.66)	230.31 (34.58)	227.62 (32.12)	223.28 (41.48)	211.82 (38.74)	205.88 (38.79)	229.05 (36.23)
**Number of frames_(Task)_**	242.18 (29.75)	247.85 (20.68)	237.46 (28.61)	234.29 (26.11)	233.41 (32.38)	215.39 (39.24)	200.44 (43.10)	233.84 (32.90)

### Fingerprint Identification Accuracy in a Lifespan Cohort

**Figure 1.**
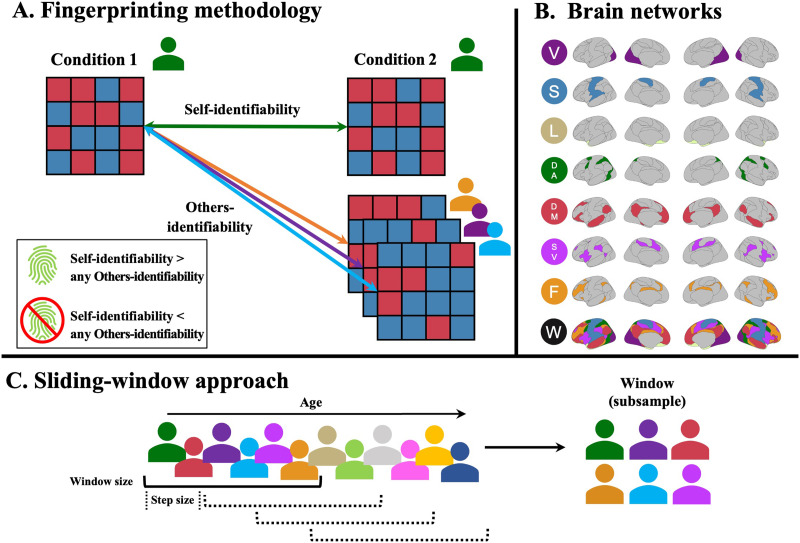
Illustration of the methodology. (A) Illustration of the fingerprinting framework. Fingerprints are established by the correlation of the functional connectivity of participants between conditions. The correlation within the same individual constitutes self-identifiability, while the correlation between individuals constitutes others-identifiability (Amico & Goñi, 2018). If the self-identifiability is higher than any other others-identifiability for a given participant, they are successfully identified (Finn et al., 2015). (B) Yeo functional networks used in the analyses of the paper. V = visual, S = somatomotor, L = limbic, DA = dorsal attention, DM = default mode, SV = salience/ventral attention, F = frontoparietal, W = whole brain. (C) Illustration of the sliding-window approach to select subgroups of participants. Subsamples of participants (window size) were chosen iteratively by taking the participants from the cohort, ordered by age, and slowly moving along (step size) the lifespan. This method yields subsets of overlapping participants across the lifespan, offering a cross-sectional, semi-continuous overview of changes during aging. Window size and step size were varied to obtain different combinations of subsamples.

To test the stability of the fingerprint metrics of interest (fingerprint identification accuracy, self-identifiability, and others-identifiability; Finn et al., 2015), we correlated the FC pattern of a given individual to their own FC pattern across Rest and Task conditions (self-identifiability) and to the FC pattern of all other individuals (others-identifiability; Amico & Goñi, 2018). If the self-identifiability correlation coefficient was stronger than any of the others-identifiability correlation coefficient, then the participant was identified as having a unique signature (fingerprint identification accuracy). This method is illustrated in Figure 1A. Edges used in the identification paradigm and the rest of the analyses comprised the whole-brain connectome, edges within a given network (within-network edges), and edges between a given network and all other network nodes (between-network edges). We used the Schaefer parcellation with 400 nodes (Schaefer et al., 2018) and Yeo’s seven-network solution (Yeo et al., 2011) to derive the FC and to compute the fingerprint metrics. Results were also replicated using the Power parcellation (264 nodes; Supplementary Figure 3A; Power et al., 2011).

**Figure 2.**
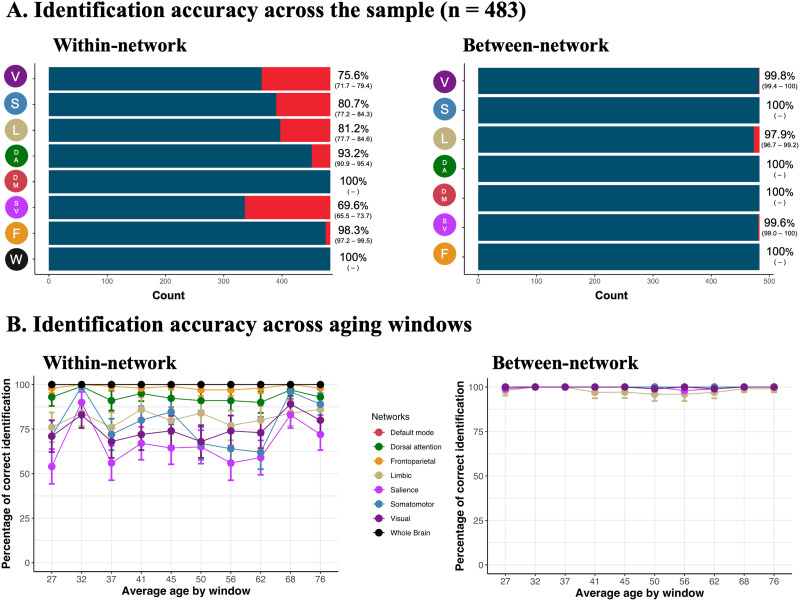
Unique connectomes across the lifespan, networks, and tasks. Fingerprint identifiability in the pair of Rest and Task conditions. Panel (A) illustrates the fingerprint identification accuracy across the entire sample using within- and between-network edges. The blue color in the bar graphs and the percentages (with confidence intervals; alpha = 0.05) to the right of the graphs indicate the proportion of individuals correctly identified. Network acronyms on the y-axes match graphics in Figure 1B and represent the specific functional network used for identification. In panel (B), we used a between-individual, age-group sliding-window approach to plot how stable the fingerprint identification accuracy was across the lifespan for each network.

Across the full sample, we found high rates—up to 100% in some networks—of fingerprint identification (Figure 2A). McNemar tests indicated that identification was increased when using between-network edges (compared with within-network edges; Supplementary Table 1) and when using higher associative cortices (default, frontoparietal, and dorsal attention compared with visual, sensorimotor, and limbic; Supplementary Table 2). Using between-network edges, we were able to achieve 100% fingerprint identification accuracy in the somatomotor, dorsal attention, default, and frontoparietal networks. Using whole-brain connectome and within-network edges in the default network, we achieved 100% fingerprint identification accuracy.

To determine how stable identifiability was throughout the lifespan, we adapted a between-participant sliding-window approach (Figure 1C; Váša et al., 2018). Briefly, participants were ordered by age, and slices of overlapping participants were selected to create groups of participants across different ages of the lifespan. Using this approach, identifiability was stable throughout the lifespan across networks (Figure 2B, Supplementary Figure 1; approach described in Figure 1C). Finally, employing McNemar tests, identification rates using within-network edges were similar for partial correlation-derived FC compared with product–moment correlation-derived FC, but were superior when using between-network edges (Supplementary Tables 3 and 4).

We also tested whether using a random collection of nodes selected throughout the brain would yield comparable fingerprint identification accuracy rather than using the nodes of the defined Yeo networks. We created two random networks by randomly selecting a subset of nodes from the Schaefer parcellation, of 22 and 91 nodes, respectively matching the size of the limbic and default networks in our study. Using the 22-node random network yielded worse accuracy than any other network at 16%, but using the 91-node random network yielded high accuracy at 94% (Supplementary Figure 2A).

### Both Self-Identifiability and Others-Identifiability Change Nonlinearly and in Parallel Across the Lifespan

**Figure 3.**
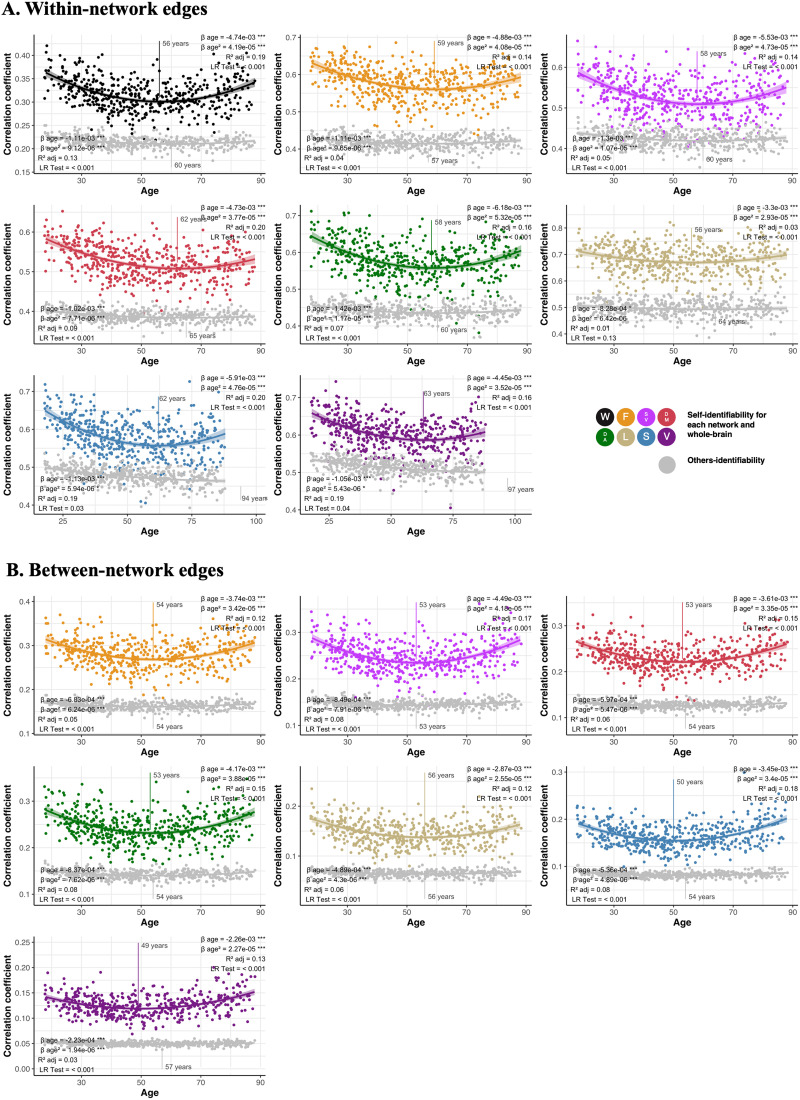
Differences in self- and others-identifiability across the lifespan. Change in self-identifiability (colors) and others-identifiability (gray) are represented using either within-network edges (A) or between-network edges (B). Each graph represents a different network, following acronyms and color schemes of Figure 1B. The beta coefficient of the age term and its quadratic term are presented at the top of the graph. We also present the adjusted *R*^2^ of the model and the *p* value of the nested likelihood ratio indicating the nonlinearity of the relationship. The *p* value of predictors surviving inclusion of covariates and execution of the bootstrapping are indicated by asterisks (***p < 0.001, **p < 0.01, *p < 0.05, °*p* < 0.10). The age at which the curve changed direction was calculated from Stimson’s equation (Stimson et al., 1978) and is illustrated on the graphs.

We found, using quadratic regressions and nested likelihood ratio tests, that both self-identifiability and others-identifiability differed nonlinearly across the lifespan across all networks (for both within- and between-network edges; Figure 3). Using Stimson’s equation for quadratic models (Stimson et al., 1978), we further found that both self-identifiability and others-identifiability appeared to decrease until 49–63 years of age, before increasing (Figure 3). Results of all models, except for others-identifiability in the limbic network, remained significant when bootstrapping and controlling for sex, handedness, motion, and number of fMRI frames available. Results were also very similar when using the Power atlas (Supplementary Figure 3B) across all networks for the self-identifiability, and for the whole-brain, default, frontoparietal, and dorsal attention networks for the others-identifiability. We also found the same results when using our two random networks described in the previous section (Supplementary Figure 2B).

To ensure that the nonlinearity of our results wasn’t driven by the oldest participants having higher self- and others-identifiability, we excluded participants above 80 years of age and repeated the analyses. We found nearly identical results (Supplementary Figure 4). Finally, we tested whether results would be similar when functional connectivity was generated using product–moment correlations of the blood oxygen level–dependent (BOLD) signal between nodes instead of using partial correlations. We found few associations between self-identifiability and age using this method. However, in networks where the association existed, it exhibited a U-shape (Supplementary Figure 5).

### Regions Contributing to Self-Identifiability Across the Lifespan

To determine which FC edges contributed to self-identifiability, we used an elastic net model paired with the age-group sliding-window approach. Specifically, we aimed to determine which combination of FC edges across the brain were predictive of self-identifiability in both a training and a testing set, in each age window. We applied these elastic net models to each age window across our sliding-window parameters. We report the model performance of the elastic net models in the left-out test set (i.e., whether edges identified in the training model also predicted self-identifiability in the left-out test set). We also report the nodal density for each node in each age window. The nodal density indicates the extent to which edges from a given node contribute to self-identifiability (Amico & Goñi, 2018). For each node, we summed the number of edges identified by the elastic net as being important for prediction within each node and divided this number by the total number of edges per node (400). While we report the results for only the window size 100 and step size 40, the results were identical across sliding-window parameters.

**Figure 4.**
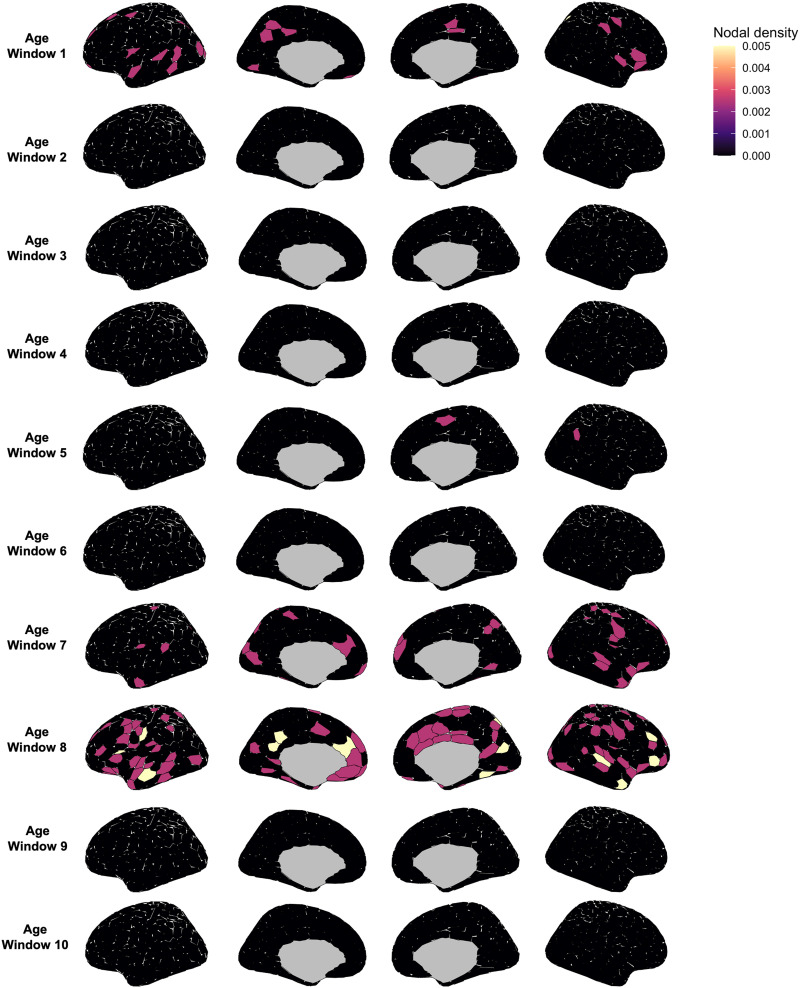
Distribution of nodes predicting self-identifiability across the brain. For each age window (see sliding-window approach, Figure 1C), we plot the nodal density (sum of number of edges identified by the elastic net as being predictive of self-identifiability divided by the total number of nodes) using the Schaefer atlas (400 nodes). A higher nodal density indicates that the node had a higher proportion of edges contributing to self-identifiability. Average age in each window matches averages in Figure 2.

Overall, prediction of self-identifiability within each age window did not generalize to any left-out samples and exhibited poor model performance across all age windows (Supplementary Figure 6). Furthermore, no specific nodes had more predictive edges than others (Figure 4). In fact, in many windows, the elastic net did not identify any combination of edges that were predictive of self-identifiability. These results suggest that no combination of FC brain edges can reliably predict self-identifiability across individuals.

A potential issue with using the elastic net model with our data is the massive number of predictors in the model (79,800 edges) and the sparse nature of the FC matrices derived from partial correlations rather than product–moment correlations. This could explain the lack of generalizability in the left-out test sets, as well as the lack of nodes identified consistently as predictive. To ensure that our results were not driven by these limitations, we adapted three more methods to verify these results: a connectome predictive modeling approach, an intraclass correlation approach, and a clustering approach. The connectome predictive modeling and intraclass correlation approaches tested whether FC in individual edges in specific networks were driving self-identifiability. Alternatively, the clustering approach aimed to confirm whether patterns of individual FC were shared across the brain in each age window. Detailed discussions of these methods are found in the Supporting Information. All these additional analyses converged toward the same conclusion as the elastic net model: Regions contributing to self-identifiability vary across the lifespan and between individuals (Supplementary Figures 7 to 14).

### Variability in FC, but Not Variability in BOLD Features, Differs Over the Lifespan

We additionally tested whether other markers of variability in fMRI signal differed over the lifespan (Supplementary Figure 15). To this end, we derived two types of measures, variability in FC and variability in BOLD temporal similarity profile. Within-individual variability in FC was defined as the variance coefficient of FC values within a given network for a given individual. We defined the between-individual variability in FC as the distance (subtraction) between the variability in FC of an individual to the average variability in FC of the group. Variability in temporal similarity profile was defined in the same way but using BOLD signal feature profiles instead of FC (Shafiei et al., 2020). More information on these measures is available in the Methods section. For both measures we calculated a within-individual variance measure and between-individual variance measure. These were calculated at the whole-brain and network levels, and for each fMRI condition (Rest and Task). In contrast to the fingerprinting results, within-individual variability in FC decreased linearly across the lifespan in all networks, and between-individual variability in FC did not change with age in any network. No associations were found between age and variability in temporal profile similarity.

Finally, we tested whether a nonlinear change in amplitude of the BOLD signal with age could be driving the nonlinear association between self-identifiability and age. Amplitude was calculated as the average of the amplitude of the BOLD signal for all nodes in a given network, for each participant. We found that there was no association between amplitude of the BOLD signal and age in the Rest modality. In the Task modality, amplitude of the BOLD signal was associated nonlinearly with age (Supplementary Figure 16), but self-identifiability was not associated with BOLD signal amplitude (Supplementary Figure 17). We conclude that it is very unlikely that the BOLD signal amplitude drives the nonlinear association between self-identifiability and age.

### Self-Identifiability Is Associated With Gray Matter Volume

Our initial results suggest that fingerprint accuracy is reliably achieved in adults over the lifespan. This is likely achieved due to the variability across people in the strongest FC patterns across the brain. We finally wanted to assess whether this identifiability was associated with age-sensitive variables, such as brain volume. To do so, we used three morphometric networks derived from an independent study as our outcome of interest (Pichet Binette et al., 2020): one frontal network (strongest age-related changes), a limbic network, which includes the hippocampus and the medial temporal lobe (moderate associations with both Alzheimer’s disease and age-related changes), and an occipital network (weakest age-related effect). Gray matter volume was extracted for all three networks and used in our analyses.

**Figure 5.**
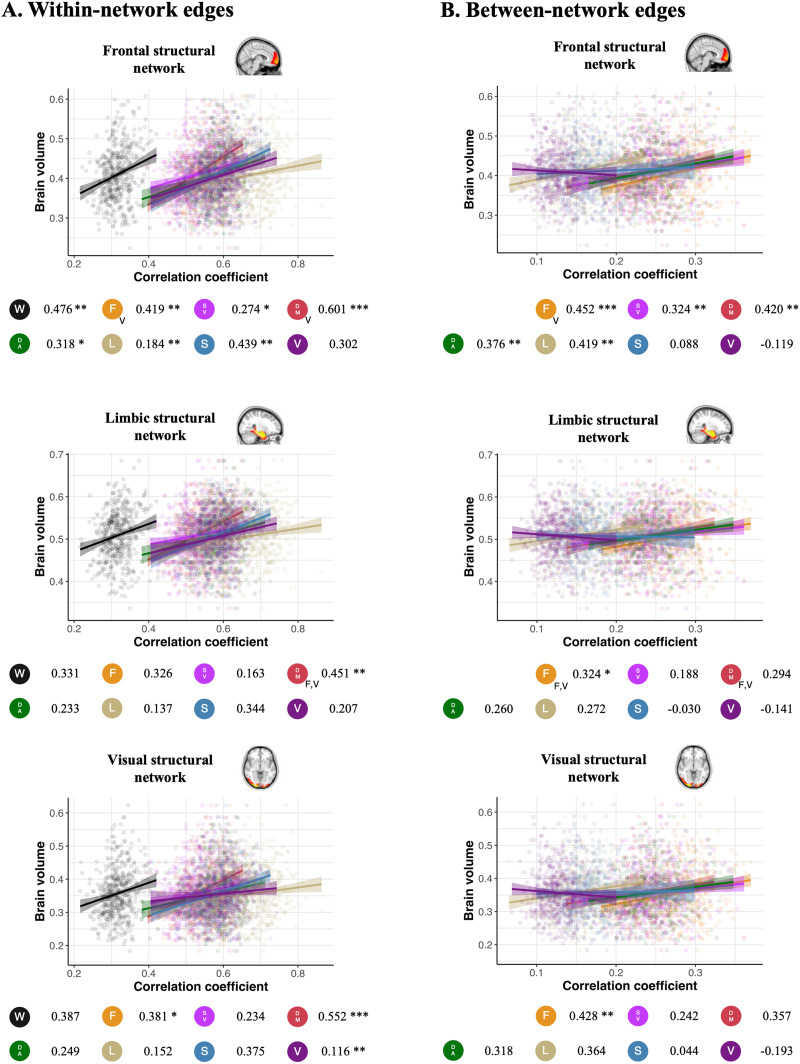
Association between gray matter volume and self-identifiability. Scatterplots presenting the association between self-identifiability, derived using (A) within- and (B) between-network edges, and gray matter volume in three gray matter morphometric networks: frontal structural network (age-sensitive network), limbic structural network (Alzheimer’s/age-related network), and visual structural network (“control” network). Data points, regression slopes, and bubbles below the graph follow the color scheme of Figure 1B. The beta coefficient of the relationship between the self-identifiability and the brain volume is indicated beside each network bubble. The *p* value of each predictor surviving comparison with covariates and bootstrapping is denoted by asterisks next to the beta coefficient (***p < 0.001, **p < 0.01, *p < 0.05). Models surviving all confounders for all three morphometric networks were compared using Vuong’s test for non-nested models. A letter at the bottom right of the network acronym indicates that the association was stronger using that specific structural network compared with the other networks referred to by the letter (V = visual, F = frontal, L = limbic).

Lower self-identifiability was associated with lower gray matter volume in the frontal structural network over and above the effect of age and other covariates (Figure 5). This connectome-wide result was recapitulated in the within- and between-network self-identifiability metrics (except for self-identifiability in the visual networks for within-network edges and visual and somatomotor networks for between-network edges).

Others-identifiability was not associated with brain volume (Supplementary Figure 18). Using our markers of variability in FC and BOLD signal, we found that decreased within-individual variability in FC was associated with decreased brain volume across networks (Supplementary Figure 19) but to a lower extent than self-identifiability. Similar to others-identifiability, there was no association between between-individual variability in FC and brain volume. We also did not find any association between variability of temporal profile similarity and brain volume.

## DISCUSSION

We found that connectome-based fingerprinting is reliable across the lifespan. Fingerprint identification accuracy was robust even though self-identifiability and others-identifiability show a nonlinear cross-sectional distribution across the lifespan. We also found that the highest weighted edges contributing to self-identifiability varied across individuals. This interindividual variability was observed within each decade of life. Furthermore, relative to whole-brain and within-network FC, between-network FC provided more reliable identification estimates and the number of nodes (and related edges) were more important than their localization to identify individuals using brain functional proprieties. Finally, we found that self-identifiability (but not others-identifiability) was associated with frontal brain volume, a morphometric feature known to atrophy with advancing age (Pichet Binette et al., 2020).

Our findings indicate that fingerprinting remains robust across the lifespan, despite observed age-related changes in FC in older adulthood (Geerligs et al., 2015; Zonneveld et al., 2019). We found high fingerprinting identification accuracy across the lifespan, with perfect to nearly perfect identifiability accuracy in the whole-brain connectomes and in the frontoparietal and default mode networks. Higher identification rates for associative cortical FC compared with unimodal networks have been found previously (Airan et al., 2016; Amico & Goñi, 2018; Finn et al., 2015; Horien et al., 2018, 2019; Van De Ville et al., 2021; Vanderwal et al., 2021) and have been suggested to be the result of high interindividual variability in FC within these regions (Geerligs et al., 2015; Mueller et al., 2013). While this might partly be the case, networks such as the default mode network include a larger number of nodes than do some unimodal networks. In supplementary analyses we showed that we could achieve almost perfect identification using a large random network composed of the same number of nodes as the ones used in the default mode network. This identification was significantly diminished in a random network that included the same number of nodes as the ones used in the limbic network. These results suggest that high identification may largely depend on the amount of information provided to perform this identification. This is in line with previous studies suggesting that finer grained parcellations—that is, more nodes—yield higher identification accuracy (Finn et al., 2015). The amount of information available for each participant might therefore be more important than the specific cortical topography when identifying individuals.

Next, we explicated fingerprint identification accuracy into its two components: self-identifiability and others-identifiability (Amico & Goñi, 2018). We found that both components have a nonlinear trajectory across the lifespan. These U-shaped trajectories comprise high scores in young adults, which decrease into middle age and increase in older adulthood. This phenomenon was present across networks, and it impacted metrics of self-identifiability more than others-identifiability. While U-shape trajectories have previously been reported in functional and structural lifespan studies (DuPre & Spreng, 2017; Kupis et al., 2021; Nadig et al., 2021), the exact cause of this phenomenon is unknown. This nonlinear trajectory could be explained by a number of factors. Interindividual heterogeneity in anatomy is most variable in early and late life, with more homogeneity observed between middle-aged adults (Nadig et al., 2021). This variance could boost individual identifiability in the youngest and oldest participants. Additionally, middle-aged women may have lower self-identifiability between fMRI sessions because of perimenopausal fluctuations in estrogen and progesterone (Pritschet et al., 2020), which impact brain function on multiple scales. Previous studies in young adults found no longitudinal change in fingerprinting accuracy over a 2- to 3-year follow-up (Ousdal et al., 2020), suggesting that a longitudinal lifespan approach is needed to better characterize age-related change occurring over decades.

We also found that change occurring with age in self-identifiability and others-identifiability mainly occurs in parallel in all networks, including the random networks, which likely explains why the high identification accuracy is preserved across the lifespan despite age-related changes in its two components. As long as the balance between self-identifiability and others-identifiability is preserved, healthy individuals can be identified among a large group of individuals with similar accuracy (Horien et al., 2019; Kaufmann et al., 2018). Findings from developmental cohorts suggest that fingerprint metrics increase rapidly from a few days after birth and stabilize in young adults (Horien et al., 2019; Hu et al., 2022; Jalbrzikowski et al., 2020; Kaufmann et al., 2018; Vanderwal et al., 2021). Additionally, neuropsychiatric symptoms lead to a decrease in self-identifiability (Kaufmann et al., 2017, 2018). From a developmental perspective, fingerprints stabilize in early adulthood, but they may be disrupted when the brain is affected by an overt neuropsychiatric or neurological disorder, or subclinical perturbations, resulting in mid-life declines in identifiability. The older adult cohort could represent either a stabilization of these effects, or a more carefully screened sample of healthy individuals that persist in the developmental stability of identification.

Our findings are consistent with work suggesting that fMRI connectivity is composed of distinct, unique individual-specific and shared task-specific components (Gratton et al., 2018; Mantwill et al., 2022). Using four different approaches (elastic net, connectome predictive modeling, edge-wise intraclass correlation, and clustering), we did not find consistent edges contributing to self-identifiability in our sample across individuals. This aligns with our large random network yielding high identification accuracy, suggesting that brain signatures, and their associated brain regions, are unique across individuals. These analytical approaches reinforce the finding that between-individual variance in functional brain connectomics are critical to the identification of individual functional fingerprints.

Finally, we aimed to determine whether individual-level FC fingerprint metrics were associated with brain volume. We compared brain volume associations with self-identifiability and others-identifiability. Whole-brain connectomes, within-network FC, and between-network FC self-identifiability were associated with brain volume in frontal areas known to be particularly affected by normal age-related atrophy (Pichet Binette et al., 2020). We also found consistent associations for self-identifiability between default and frontoparietal networks and brain volume in the hippocampus and medial temporal lobe (limbic structural network; known to be vulnerable in Alzheimer’s disease). The occipital structural regions were preserved over the lifespan and did not impact self-identifiability (Pichet Binette et al., 2020). In contrast, there was no consistent association between others-identifiability and brain volume.

Self-identifiability may be associated with a number of factors that differentially impact individuals over the lifespan (Kaufmann et al., 2017, 2018). For instance, the default network is affected by a wide range of neurological disorders (de Lange et al., 2019) and aging (Zonneveld et al., 2019). Consistent with this idea, reduced fingerprint identifiability within the default network has been associated with mental health disorders in youth (Kaufmann et al., 2017) and reduced brain volume in older adults (Ousdal et al., 2020). Individual-level FC measures, rather than group-level measures, may be more appropriate to detect age- or disease-related functional changes (Finn & Constable, 2016), either by directly accounting for interindividual differences (Finn & Rosenberg, 2021) or, at least partially, by ignoring group-level noise in fMRI signal (Amico & Goñi, 2018). Targeting individual-level differences might be particularly important in aging research, as aging individuals present high diversity of lifestyle and medical history, leading to diverse age-related outcomes (Daskalopoulou et al., 2019).

As an additional note, the findings above also expand methodological aspects in the field of fingerprinting. First, we used partial correlation to generate FC matrices, which were then used for fingerprinting. Partial correlations resulted in higher identification rates in our study compared with product–moment correlations. Partial correlations produce sparse matrices excluding, to some extent, noise inherent to fMRI (Marrelec et al., 2006). Other methods aiming at removing noise in FC matrices have also found success in improving fingerprint identification accuracy (Amico & Goñi, 2018). As noise affects individuals across the sample in a similar manner (Amico & Goñi, 2018), removing noise should therefore better isolate individual-specific features. Second, we computed identification using between-network edges as well as within-network edges. In most cases, between-network edges provided better identification rates compared with within-network edges. To our knowledge, all studies on fingerprinting to date have used within-network edges to compute identification. However, while within-network edges represent communities of nodes working tightly together (Sporns & Betzel, 2016), between-network connections are necessary to various brain functions. In aging in particular, the segregation of these networks tend to change (Chan et al., 2014), and as such between-network edges may better represent overall brain network communications. Our study therefore also suggests potential methodological aspects to consider in future fingerprinting studies, particularly when considering aging populations.

### Strengths and Limitations

The main limitation of this study is its cross-sectional nature. As such, it is difficult to determine how fingerprint metrics change during the lifespan within individuals, limiting the interpretation of our conclusions. Future work would benefit from longitudinal studies examining identifiability across scans separated by many years. Similarly, because of quality control issues, the eldest participants in our cohort were underrepresented, and as such, the nonlinearity of our findings need to be interpreted with caution. However, supporting the nonlinearity of the findings, the nonlinearity between age and self-identifiability was still preserved when removing the oldest participants. Finally, we included only two fMRI modalities in our analyses. As other studies have shown that the choice of the task can influence the ability to fingerprint, future studies should replicate our findings using other combinations of fMRI tasks.

### Conclusions

Across our analyses, we found that FC patterns allow for precise fingerprinting between individuals and that this discrimination is reliable across the lifespan. High identification rates were observed across the Cam-CAN cohort, accompanied by age-related effects on individual-level (self-identifiability) and group-level (others-identifiability) FC patterns. Accurate fingerprinting of FC was observed across the lifespan, even though edges contributing to self-identifiability differed between individuals across networks and across the decades of life. Finally, we show that individual-level self-identifiability (instead of group-level others-identifiability) is associated with brain volume in regions vulnerable to age-related atrophy. Together, the present findings illuminate the potential utility of individual-level measures that demarcate age-related brain change. Group-level differences in FC have revealed reliable patterns attributed to the aging brain. However, individual differences in FC patterns are likely to play a key role in predicting brain health and associated functional outcomes.

## MATERIALS AND METHODS

### Participants

Data used in the preparation of this work were obtained from the Cam-CAN repository (available at https://www.mrc-cbu.cam.ac.uk/datasets/camcan/). The Cam-CAN cohort is a lifespan cross-sectional population-based cohort, composed of cognitively healthy participants aged between 18 and 89 years of age residing in the United Kingdom. Full details for the study participants and recruitment can be found elsewhere (Shafto et al., 2014; Taylor et al., 2017). Participants underwent several brain imaging procedures at one time point. Special attention was given to recruiting persons from different decades and to balance both men and women. This study was approved by the Cambridgeshire 2 Research Ethics Committee (reference: 10/H0308/50).

### Magnetic Resonance Imaging

Full details on the imaging data collection and on the fMRI tasks used are available elsewhere (Taylor et al., 2017). Briefly, MRI data were acquired on a 3T Siemens TIM Trio scanner with a 32-channel head coil for a 1-hr session. T1-weighted MPRAGE sequences were acquired for structural imaging, and T2*-weighted EPI sequences were acquired for fMRI imaging (261 volumes with 32 axial slices each; slice thickness of 3.7 mm; interslice gap of 0.74 mm; TR = 1,970 ms; TE = 30 ms; flip angle = 78 degrees; FOV = 192 mm × 192 mm; voxel size = 3 mm × 3 mm × 4.44 mm). Participants underwent two different fMRI conditions during one session: a resting-state condition (Rest), where participants were asked to keep their eyes closed for 8 min, 40 s; and a sensorimotor task (Task), also of 8 min, 40 s, in which participants were asked to press a button when audiovisual stimuli were presented.

Functional images from the two modalities were preprocessed using the NeuroImaging Analysis Kit, version 0.12.4 (NIAK; https://niak.simexp-lab.org/; Bellec et al., 2011; Gonneaud et al., 2020). The first three volumes of each run were suppressed to allow the magnetization to reach equilibrium. Images were slice-timing corrected, and rigid-body motion parameters were estimated for each time frame. For registration, T1-weighted images were linearly and nonlinearly registered to MNI space. The rigid-body transform, fMRI-to-T1 transform, and T1-to-stereotaxic transform were all combined, and the functional volumes were resampled in the MNI space at a 3-mm isotropic resolution. To account for potentially excessive motion, frame displacement was calculated for each volume, and those with more than 0.5 frame displacement were removed with one prior adjacent frame and two consecutive frames after. Time series with less than 40% of their original data after removing excessive motion were discarded from subsequent analyses (Orban et al., 2015). Next, slow time drifts, cerebrospinal fluid, average white matter signal, and motion artifacts (first principal components of the six rigid-body motion parameters, and their squares) were removed from the fMRI time series, and fMRI volumes were smoothed with a 6-mm Gaussian kernel. Finally, fMRI time series for each region of the Schaefer atlas (*n* = 400) were extracted using Nilearn 0.6.2 (Abraham et al., 2014). Partial correlations were used to generate FC between regions, accounting for the signal in all other brain regions. This process generates sparser functional connectivity matrices that are thought to account for more noise than using simple product–moment correlation and represent more direct connections between regions (Marrelec et al., 2006; Zalesky et al., 2012). We generated FC matrices for the task and resting-state fMRI runs.

All structural images were preprocessed using Statistical Parametric Mapping (SPM12, https://www.fil.ion.ucl.ac.uk/spm/software/spm12/) in MATLAB version 2012a, as part of a previous study (Pichet Binette et al., 2020). Images were segmented into gray matter, white matter, and CSF components. Then, a group-specific template was created using the Diffeomorphic Anatomical Registration through Exponentiated Lie Algebra toolbox (DARTEL; Ashburner, 2007), which was then registered nonlinearly to the MNI-ICBM152 template. Finally, each individual participant’s gray matter map was registered back to the group template, before being smoothed with an 8-mm^3^ isotropic Gaussian kernel.

### Sliding-Window Analysis

Across multiple analyses, we used a between-participant sliding-window approach to study differences across the lifespan in a semi-continuous manner (Váša et al., 2018; Figure 1C). First, participants were ordered by age (from youngest to oldest). Then, we iteratively selected subsamples of overlapping participants varying two parameters: window size (i.e., the number of participants in each subsample) and step size (i.e., the number of participants skipped before selecting the next window). We used window sizes of 100 or 150 participants and used a step size of either 25 or 40 participants. As such, participants in adjacent windows overlapped by 60% to 80%. Main analyses report results using a window size of 100 and step size of 40, while results using other parameters are reported in supplementary analyses.

### Fingerprinting

Our main interest was functional connectome fingerprinting, which encompasses three measures of interest: fingerprint identification accuracy (Finn et al., 2015), self-identifiability, and others-identifiability (Amico & Goñi, 2018). In the fingerprinting framework, the FC matrix of a given individual obtained from one fMRI condition is correlated to the FC matrix of the same individual obtained during a different fMRI condition by computing the correlation coefficient of the vectorized upper triangle z-values between sessions. This results in a within-individual correlation (i.e., self-identifiability). This process is repeated for all within- and between-participant FC matrices, permitting the computation of both within- and between-individual similarities (i.e., others-identifiability). Finally, fingerprint identification accuracy is estimated from both self- and others-identifiability measures: A fingerprint is considered identifiable when self-identifiability exceeds the magnitude of others-identifiability (Amico & Goñi, 2018; Finn et al., 2015; see Figure 1A for schematic overview). This process was done for the whole-brain connectome as well as for within-network and between-network edges for each network.

The fingerprint framework was adapted from the original methodologies by Finn et al. (2015) and Amico and Goñi (2018). The FC obtained for the Rest and Task modalities were first normalized using Fisher’s r-to-z transform. Product–moment correlations were then used to correlate FC matrices obtained from the Rest and Task conditions, deriving self-identifiability and others-identifiability. These values where then stored in a similarity matrix. For each row of the matrix, a fingerprint identification was considered accurate if self-identifiability (diagonal elements of the matrix) was higher than any other others-identifiability (off-diagonal elements of the matrix). Self-identifiability and others-identifiability were computed based on the FC between 400 parcels. Additionally, the parcel information corresponding to the Yeo seven networks was leveraged to demarcate within-network FC. Then, we composed aggregates of between-network FC by taking any edges between each other network. For example, if the visual network was composed of nodes 1 to 61 (rows and columns 1 to 61 in the matrix), within-network edges comprised edges where both rows and columns were between 1 and 61. Between-network edges comprised edges in rows 1 to 61 but in columns 62 to 400 (Yeo et al., 2011).

Additionally, we created two random networks to test whether we could find high identification accuracy using edges belonging to a random assortment of nodes rather than using predefined networks. We selected two random sets of nodes across the brain. The number of nodes chosen were 22 and 91, to match the size of the smallest (limbic) and largest (default) networks (Schaefer et al., 2018; Yeo et al., 2011) included in our study. Fingerprinting methodology described above was applied to the edges of these two random networks.

### Predicting Self-Identifiability Using Combinations of Edges

We adapted an elastic net approach to predict self-identifiability from the FC edges in our sample. Specifically, we used the sliding-window method to select subsamples of participants across the lifespan. Within each window, we first removed the diagonal and lower triangle of the functional connectivity matrices of the participants and flattened the remaining 79,800 edges. The connectivity values were then standardized, and participants were randomly split in a training and testing subset (85% training, 15% testing). Connectivity values across all 79,800 edges were used to predict self-identifiability. In the training set, we used a 5 k-fold cross-validation with a grid search to select the optimal L1 ratio for our elastic net model. Once the optimal L1 ratio was selected, an elastic net model was fitted on the entire training set. The model was then used to predict self-identifiability in the testing set. Performance in the testing set was reported as the variance explained (*R*^2^) and the root mean square error (RMSE).

The elastic net outputs coefficients indicating which edges significantly contributed to model performance. To determine their topography in the brain, we adapted a nodal density approach (Amico & Goñi, 2018). Briefly, edges were resized to a 400 × 400 functional connectivity matrix format and binarized. Then, the sum of the binary coefficients for each node (i.e., each row of the matrix) was divided by the number of edges for that node (i.e., 400). This yields a nodal density measure for all 400 nodes of the Schaefer atlas, where more density indicates that edges in that node are more important. These results were then projected to a brain map.

### Age-Related Outcome: Structural Aging Morphometric Network

We tested the relationship between functional fingerprint metrics (self-identifiability and others-identifiability) and structural age-related changes. Three morphometric networks derived in an independent study were used as our outcome of interest (Pichet Binette et al., 2020). Briefly, a large cohort of cognitively unimpaired younger and older adults and participants on the Alzheimer disease spectrum were grouped and independent component analysis was used to derive statistically independent structural brain networks. Then, volume in each component was used in a receiver operating characteristic analysis to determine whether brain volume could accurately classify individuals in their corresponding group. From this study, we chose one frontal network (ICA01 in the original study, which was the network showing the strongest age-related changes), a limbic network (ICA10, which showed moderate associations with both Alzheimer’s disease and age-related changes), and an occipital network (ICA15, which showed the weakest age-related effect). Gray matter volume was then extracted for all three morphometric networks for each participant. These morphometric networks were chosen rather than using parcel-level or network-level gray matter measures, as we wanted age-associated gray matter measures and as their association with fingerprint measures could be more directly comparable between networks.

### Supplementary Analyses on FC Variability

Additionally, we also determined whether other markers of variability in fMRI signal changed during the aging process. We derived two types of measures, variability in FC and variability in BOLD temporal similarity profile.

Both types of measures were calculated using custom Python scripts. Variability in FC for each individual was computed as the variance coefficient of FC coefficients at a whole-brain level and within each network of interest. To obtain between-individual variability, we first computed the average variability in FC across the sample. Then, for each individual, we computed the absolute distance between their variability and the mean variability of the group. A greater distance indicates more between-individual variability.

Variability within and between individuals in BOLD signal features was computed as the variance in temporal profile similarity of BOLD signal features (Shafiei et al., 2020). To do so, BOLD signal features were extracted from each brain region using the highly comparative time series analysis toolbox (Fulcher & Jones, 2017; Fulcher et al., 2013). Time series were first z-scored and then fed to the toolbox where 7,700 features were extracted from the BOLD signal. Because the number of fMRI frames varied between individuals after the preprocessing, some features had missing or constant values across participants and were therefore dropped. This resulted in a final 6,192 features remaining. Extracted features were then z-scored again to ensure comparability between features. For each participant, the extracted features time series for each region were correlated using product–moment correlations to determine how similar time series were between each brain region, resulting in a 400 × 400 temporal similarity profile similarity matrix. Finally, variability in temporal similarity profile was computed identically to the variability in FC.

As a final verification, we computed BOLD signal amplitude and related this measure to age and self-identifiability. In each of the 400 brain regions, for each participant, the amplitude of the time series were computed by subtracting the minimum from the maximum BOLD value. Then, the amplitude of the nodes within the network was averaged to obtain a single amplitude measure for each network for each participant.

### Software

The sliding-window, fingerprinting framework, elastic net, edge-wise intraclass correlation analyses, connectome predictive modeling, clustering, and variability in FC analyses were adapted and developed using Python 3.8.5 (Python Software Foundation, https://www.python.org/; NumPy 1.19.1, Harris et al., 2020; pandas 1.1.3, McKinney, 2010; Pandas Development Team, 2020; scikit-learn 0.24.0, Abraham et al., 2014; SciPy 1.7.1, Virtanen et al., 2020; pingouin 0.5.2, Vallat, 2018) on Béluga, a high-performance computing resource hosted by the Digital Research Alliance of Canada, running on CentOS 7.9. The temporal similarity profile analysis was done using MATLAB 2021b (highly comparative time series analysis toolbox; Fulcher & Jones, 2017; Fulcher et al., 2013) and Python 3.8.5. All statistical analyses and graphs were done in R v4.1.2 (R Core Team, 2020; tidyverse 1.3.0, Wickham et al., 2019; boot 1.3-28, Canty & Ripley, 2021; Davison & Hinkley, 1997; patchwork 1.1.1, Lin Pedersen, 2020; ggnewscale 0.4.8, Campitelli, 2022; ggseg 1.6.5, Mowinckel & Vidal-Piñeiro, 2020) using R Studio (“Ghost Orchid” Release [077589bc, 2021-09-20]) for macOS Monterey 12.6. All code related to the analyses are available at https://github.com/villeneuvelab/projects (St-Onge, 2022). The code related to the fingerprinting analysis was also adapted to the openly available *sihnpy* Python package (https://sihnpy.readthedocs.io/).

### Statistical Analyses

Fingerprint identifiability was computed for all networks for the Rest and Task conditions for both within- and between-network edges. Percentage of identified individuals with confidence intervals were calculated. Paired McNemar tests were used to compare identification in a given network to the identification of the same network using a different fingerprint type (i.e., using within- vs. between-network edges). We used this approach to compare network performance in fingerprint identifiability within each modality pair (e.g., comparing proportion of identification using the default vs. somatomotor networks). A family-wise Bonferroni correction was applied to each set of comparisons to account for multiple comparisons. Finally, we used the sliding-window approach where the fingerprint identification accuracy in each age window was computed and visualized.

To study the relationship between self-identifiability and age, and others-identifiability and age, we used multiple linear regression models. We used polynomial (quadratic) regressions when the linearity assumption was violated and, in that case, used Stimson’s equation to derive the peak’s coordinates (Stimson et al., 1978). Assumption violation was determined by comparing fitted to residual values. We also used nested likelihood ratio tests to test whether models using quadratic terms outperformed linear models on model fit to confirm the nonlinearity of the relationship. Each model included common confounders known to affect either FC or fingerprints as covariates of no interest: the number of frames post-scrubbing (Amico & Goñi, 2018; Horien et al., 2018; Xu et al., 2016), the mean frame displacement (i.e., movement; Amico & Goñi, 2018; Geerligs et al., 2017; Guo et al., 2012; Horien et al., 2018; Jalbrzikowski et al., 2020), the sex of participants (Finn et al., 2017), and a continuous handedness measure (Bailey et al., 2020). One model was generated for each network for both self-identifiability using within- and between-network edges (45 models total). The same number of models was generated to study the association between others-identifiability and age. Because of the high number of regressions, we applied a bootstrap resampling procedure to each model as a way to account for multiple comparisons (Westfall, 2011). Specifically, we generated bootstrapped bias-corrected and accelerated confidence intervals for the β-coefficients (where coefficients not overlapping with 0 were considered significant) of the main exposure. All analyses were repeated using within- and between-individual variability in FC and BOLD signal (i.e., variability in FC and variability in temporal similarity profiles) in each fMRI task in each network and within the whole brain.

All procedures described above were reapplied to study the relationship between fingerprint metrics and brain volume. Individual variability in FC and temporal similarity profile were also associated with age and with gray matter volume using the methodology described above. These metrics were used to determine whether other fMRI measures would also show interindividual variability across the lifespan. To test whether the association was stronger in the frontal structural network, the limbic structural network, or the visual structural network, we used Vuong’s test for non-nested models (Vuong, 1989). Models were repeated including all covariates with and without age.

## ACKNOWLEDGMENTS

The authors would like to thank Jonathan Gallego Rudolf, Yara Yakoub, Ting Qiu, and Julien Menes for useful feedback on the project, and Gabriel St-Onge for technical help in implementing the connectome predictive modeling. We would also like to thank the reviewers for providing their time to review this paper and for sharing their thoughtful insights into our results, which significantly strengthened the paper. Data collection and sharing for this project were provided by the Cambridge Centre for Ageing and Neuroscience (Cam-CAN). Computations were enabled in part by support from Calcul Québec (calculquebec.ca) and the Digital Research Alliance of Canada (alliancecan.ca), who provided access to the Béluga high-performance computing resource. This research was undertaken thanks in part to funding from the Canada First Research Excellence Fund and Fonds de recherche du Québec, awarded to the Healthy Brains, Healthy Lives initiative at McGill University. The funding organizations did not have a role in writing the manuscript.

## SUPPORTING INFORMATION

Supporting information for this article is available at https://doi.org/10.1162/netn_a_00320.

## AUTHOR CONTRIBUTIONS

Frédéric St-Onge: Conceptualization; Data curation; Formal analysis; Methodology; Software; Visualization; Writing–original draft; Writing–review & editing. Mohammadali Javanray: Conceptualization; Formal analysis; Writing–review & editing. Alexa Pichet Binette: Conceptualization; Methodology; Writing–review & editing. Cherie Strikwerda-Brown: Conceptualization; Writing–review & editing. Jordana Remz: Conceptualization; Software; Writing–review & editing. R. Nathan Spreng: Methodology; Supervision; Writing–review & editing. Golia Shafiei: Methodology; Writing–review & editing. Bratislav Misic: Methodology; Supervision; Writing–review & editing. Étienne Vachon-Presseau: Methodology; Supervision; Writing–review & editing. Sylvia Villeneuve: Conceptualization; Funding acquisition; Methodology; Resources; Supervision; Visualization; Writing–original draft; Writing–review & editing.

## FUNDING INFORMATION

Cam-CAN, UK Biotechnology and Biological Sciences Research Council, Award ID: BB/H008217/1. Frédéric St-Onge, Healthy Brains, Healthy Lives through the Canada First Research Excellence Fund and the Fonds de Recherche du Québec, Award ID: NA. Frédéric St-Onge, Mitacs (https://dx.doi.org/10.13039/501100004489), Award ID: NA. Frédéric St-Onge, Fonds de Recherche du Québec–Santé (https://dx.doi.org/10.13039/501100000156), Award ID: 316992.

## TECHNICAL TERMS

Functional connectome fingerprinting: Framework used to test how unique individuals’ patterns of functional connectivity are.
Fingerprint identification accuracy: If an individual’s self-identifiability is higher than any others-identifiability, fingerprint identification is accurate.
Self-identifiability: Correlation (i.e., similarity) between the pattern of functional connectivity of a single individual across fMRI conditions.
Others-identifiability: Average of the correlation (i.e., similarity) between the pattern of functional connectivity of an individual to all other individuals in the cohort across fMRI conditions.
Edge: One functional connectivity edge represents the functional connection between two nodes.
Elastic net: Machine learning method used to predict which combination of predictors is associated with an outcome.
Node: A parcel representing a single region from a brain atlas. Multiple nodes compose a network.
Sliding-window approach: Sample selection method in which participants are ordered by a variable (here, age) and iteratively selected to create overlapping subsamples.
Nodal density: Sum of number of edges contributing to an outcome within a node, divided by the number of edges in the node.
Temporal similarity profile: Correlation of a wide range of BOLD signal features between each node, yielding how similar the features of a node are to the features of a different node.

## Supporting Information

https://github.com/villeneuvelab/projects
