# Supplementary material for: Functional connectome fingerprinting across the lifespan
Source: Netw Neurosci. 2023 Oct 1;7(3):1206–27. doi: 10.1162/netn_a_00320 (PMC10473304; doi:10.1162/netn_a_00320)
Supplement: Supplementary file 1 [file netn-7-3-1206-s001.pdf]

Functional connectome fingerprinting across the lifespan – Supplementary materials

Frédéric St-Onge[1,2], Mohammadali Javanray[1,2], Alexa Pichet Binette[3], Cherie Strikwerda-Brown[2], Jordana Remz[2], R. Nathan Spreng[4], Golia Shafiei[5], Bratislav Misic[5], Étienne Vachon-Presseau[6,7,8], Sylvia Villeneuve[1,2,5]

1. Integrated Program in Neuroscience, Faculty of medicine, McGill University, Montreal, Canada
2. Research Center of the Douglas Mental Health University Institute, Montreal, Canada
3. Clinical Memory Research Unit, Faculty of Medicine, Lund University, Lund, Sweden
4. Department of Neurology and Neurosurgery, Montreal Neurological Institute, McGill University, Montreal, Canada
5. McConnell Brain Imaging Centre, Montreal Neurological Institute, McGill University, Montreal, Canada
6. Faculty of Dental Medicine and Oral Health Sciences, McGill University, Montreal, Canada
7. Department of anesthesia, Faculty of medicine, McGill University, Montreal, Canada
8. Alan Edwards Centre for Research on Pain (AECRP), McGill University, Montreal, Canada

Corresponding Authors:

Sylvia Villeneuve
Douglas Mental Health University Institute
Perry Pavilion Room E3417.1
6875 Boulevard LaSalle
Montreal, QC
Canada H4H 1R3
Phone: 514 761-6131 ext.: 3960
E-mail: Sylvia.villeneuve@mcgill.ca

&

Frédéric St-Onge
Douglas Mental Health University Institute
Perry Pavilion Room E3417.1
6875 Boulevard LaSalle, Montreal, QC
Canada H4H 1R3
E-mail: Frederic.st-onge@mail.mcgill.ca

Supplementary methods

Identifying regions contributing to fingerprinting metrics

a. Connectome predictive modelling

We tested whether edges most associated with fingerprint metrics changed during the lifespan by adapting a connectome predictive modelling approach. (Shen et al., 2017) Procedures are illustrated in Supp Fig 7 and can be divided in three major sections: I) Sample selection, II) Cross-validation and III) Generalizability.

In the sample selection section, we used the sliding-window approach described in the methods section of the manuscript. Once windows were derived, each window was split in a training and testing set, where 85% of the participants were retained for training and 15% for testing the generalizability of the model. (Varoquaux et al., 2017)

During the cross-validation section, we used a leave-one-out procedure (1) (Supp Fig 7C). (Scheinost et al., 2019) In each iteration, each of the 160,000 FC edges for participants in the training set were correlated to their measured fingerprint metrics of interest (self-identifiability or others-identifiability) using product–moment correlations (2). Edges with significant ($p < 0.01$) negative (lower FC associated with higher fingerprint metric) and positive (higher FC associated with higher fingerprint metric) correlations ($p < 0.01$) were kept and considered as potential predictors of the fingerprint metric of interest (3). The fingerprint metric of interest was calculated at the whole-sample level, and not within individual sliding windows. We then created two binary masks at the whole-brain level; one including significant edges from positive correlations and the other including significant edges from negative correlations (4). After discarding the lower triangle of the binary masks (symmetric projection of the upper triangle), FC values in the significant edges were then summed to obtain a single value per participant for each mask (5). These sums were used to train the models to predict the fingerprint metric of interest (6). We used two different

algorithms to train the models: a simple linear regression and a support vector regression with radial basis function kernel. For the support vector regression, both the feature and outcome were scaled to a mean of 0 and standard deviation of 1 before training the model. Hyperparameters (C, gamma and epsilon) for the final model were determined during cross-validation using a grid search technique. The positive and negative masks were then applied separately to the left-out participant (7) and their FC sum (8) was used to validate the models (9). As a last step we developed a positive and a negative cross-validated binary mask, where edges that were significant across 95% of the cross-validation runs were kept (10), and the rest was discarded. This process was done to identify FC edges that were recurrently predictive across participants of a given age group bin.

In the generalization section, we tested the predictive value of the positively associated and negatively associated models using the corresponding cross-validated binary masks in the test set (i.e., participants that were not used to develop the predictive models). The FC values in the cross-validated binary masks were summed for each participant of the test set (11) and used to predict the fingerprint metrics (12). Model generalizability in the test set was assessed by correlating the predicted and measured fingerprint metrics in the test set participants and by measuring the root mean square error.

Finally, we used the averaged cross-validated positive and negative binary masks to determine which inter-regional FC values were associated with our fingerprint metrics across the lifespan using the between-individual sliding window approach. Specifically, we divided the masks for each subsample in within- and between-network edges for each of the Yeo networks. Then, we calculated the proportion of predictive edges in each network by dividing the number of

predictive edges over the total number of edges in the network. These proportions were then plotted for visualization.

b. Edge-wise intraclass correlation

We adapted the edge-wise intraclass correlation (ICC) methodology from Amico and Goñi (2018) (see Supp Fig 10) to determine which edges contributed to self- and others-identifiability across the lifespan. In our context, we refer to *intra-rater reliability* ICC measures (as opposed to inter-rater reliability); i.e., how similar a score is for different targets within each rater. (Shrout & Fleiss, 1979) Specifically, we use the ICC form referred to as ICC(3,1) in the original publication (Koo & Li, 2016; Shrout & Fleiss, 1979) for single fixed raters. We consider two types of raters. For the first type, we consider each participant as a rater, and we consider targets as the FC of a given edge for both modalities. In this analysis, the ICC reflects how consistent the value of the edge is between the Rest and Task sessions for each participant. When the edge values of both tasks are close to one another (i.e., the difference between the two values is small), across all raters, then the ICC value for an edge would be high. Conversely, if the edge values are very different between the Rest and Task sessions across all raters, then the ICC value for an edge would be low. This first ICC measure relates to self-identifiability. While self-identifiability is derived from correlating the entire functional connectivity of a given network during rest to the entire functional connectivity of the same network during the task, edges with consistent values (i.e., high ICC) between Rest and Task are likely to contribute to higher self-identifiability due to their similarity in each participant. For the second intra-rater ICC, we set the raters as the fMRI conditions and the targets as the FC of the edge of individual participants within each condition. In this analysis, the ICC reflects how consistent the value of an edge is across participants within each modality. If the value of the edge is consistent across all participants in both the rest and task conditions

individually, the resulting ICC will be high. Conversely, if the edge value differs between participants in each modality, this would result in a low ICC. This second ICC measure relates to others-identifiability. Others-identifiability is computed by correlating the functional connectivity of one participant to the functional connectivity of a different participant. As such, if an edge has consistent values across participants in each modality, it will likely result in a higher others-identifiability. This process was done for every age window, and FC edge, yielding two matrices of 160,000 edges per age bin. The lower triangle was ignored for the rest of the analyses.

Once edge-wise ICC were derived, we first thresholded the coefficients to retain the edges contributing most to self- or others-identifiability by using values above the 95th percentile. Then, we computed the mean and standard deviation of the ICC for both within- and between-network FC. Finally, the ICC in each edge retained after thresholding was binarized: values above the 95th percentile were set to one and the rest were set to zero. The mean and standard deviation of the ICC values were then plotted for each age window.

Then, we aimed to determine whether edges identified as contributing most to self- and others-identifiability would remain the same through the lifespan. To do so, we used the binarized matrices of the ICC and the Jaccard Similarity Index (Jaccard, 1912). The Jaccard Similarity Index measures the size of the intersection of two binary arrays over their union, yielding a score ranging from zero to one, where one is a perfect overlap between the two arrays. We applied this measure by comparing the overlap of the binary arrays between each pair of adjacent age group bins (e.g., the binary array of the youngest group compared to the binary array of the second youngest group). Higher values indicate the highest weighted edges that predict identifiability overlap between adjacent groups. We also applied this method to compare the overlap between the binary arrays of the edges contributing most to self-identifiability and the binary arrays of the edges contributing

most to others-identifiability. As the Jaccard Index represents a proportion of overlap by network, we can compute a 95% confidence interval of that proportion, by calculating the standard deviation of the proportion and multiplying it by the desired degree of confidence.

Finally, we checked whether certain nodes have edges which contribute predominantly to self- and others-identifiability. Based on methodology by Amico & Goñi (2018), we computed the nodal density of each node in the binarized ICC matrix. Briefly, each row of the matrix was summed, ignoring the diagonal. Then, the sum was divided by the number of edges. This yields a nodal density measure for all 400 nodes of the Schaefer atlas. These results were then projected onto a parcellation.

c. Clustering

To determine if specific edges overlapped between individual- and group-level FC patterns, we adopted a K-means clustering approach. Specifically, the FC edges of the upper triangle of FC matrix (79,800 edges) of all participants within each age window were averaged across participants to obtain a group-level average of FC. Then, we used a K-means clustering approach to cluster the group-level average edges. We chose a 7-cluster solution to match the number of expected networks from the Schaefer parcellation used throughout the paper. The labels assigned by the clustering algorithm for each edge were saved. Then, we repeated the procedure but this time at the individual-level; we applied a 7-cluster K-means clustering algorithm to cluster the 79,800 edges of each participant. Finally, we computed the overlap of individual- and group-level clusters using the Jaccard coefficient. Specifically, for each cluster (from 1 to 7), we computed the average overlap between individual participants and the group-level FC average, where ‘1’ indicates a perfect overlap of the cluster assignments and ‘0’ indicates poor overlap of the cluster assignments.

Supplementary results

Regions predictive of fingerprint metrics

a. **Connectome Predictive Modelling**

As a complementary analysis, we used a connectome predictive modelling approach (Shen et al., 2017) to determine if any individual FC edges were predictive of self-identifiability and others-identifiability across adjacent age windows, across different sliding-window parameters (Supp Fig 8 and 9).

The connectome predictive modelling approach (Shen et al., 2017) first selected edges most associated with self-identifiability using a leave-one-out cross-validation after holding out 15% of the sample, and FC in the remaining associated edges were used to train linear and support vector regression models predicting self-identifiability. This procedure was done in each age window separately. Edges associated with self-identifiability in 95% of the cross-validations in each age window were deemed as contributing to self-identifiability. Prediction within each age window did not generalize to left-out samples, and no single network had more predictive edges for self-identifiability. Using a random set of edges instead of doing feature selection also yielded similar results (not shown). Predicting the self-identifiability across the entire sample, instead of within each age window, yielded similar results (not shown).

b. **Edge-wise intraclass correlation**

We used an intra-class correlation (ICC) analysis (Supp Fig 10), as proposed by Amico and Goñi (2018), paired with the age-group-bin sliding window approach (Figure 1B) to assesses intra-rater reliability. Specifically, we measured to which extent the connectivity values in each edge were consistent within each individual across the Rest and Task condition. High consistency (i.e., higher ICC) indicated that the edge contributed to self-identifiability within each participant.

We also measured to which extent the connectivity values in each edge were consistent within each fMRI condition across participants. In this case high consistency (i.e., higher ICC) indicated that the edge contributed to others-identifiability in each fMRI modality. This process yielded two ICC values for each edge. Additional explanation on how the intra-rater reliability was derived is available in the supplementary methods section. The sliding window approach was used to estimate whether the top 95% edge-weights in each window remained consistent across the lifespan. Within each age window, we thresholded the ICC values at the 95th percentile to retain edges contributing the most to each of self- and others-identifiability.

Thresholded ICC values indicated that edges contributing the most to self-identifiability were not consistent across individuals as they showed very low ICC values despite retaining only the highest 95th percentile edge values (i.e., poor consistency within raters between Rest and Task modality), while edges contributing the highest to others-identifiability were more consistent across individuals (i.e., high consistency within modalities between individual participants; Supp Fig 11A). This was observed across all age windows, across the whole brain connectome, within- and between-networks, and using different sliding window parameters (i.e., size of subsamples and number of participants from adjacent age-group bins). We did not find evidence that edges within or between any one network were more similar across individuals for edges contributing to self- or to others-identifiability (overlapping confidence intervals). Similar results were observed using different window parameters (not shown).

Next, we evaluated whether the edges contributing the highest to self- and others-identifiability were similar between subsamples across the lifespan (Supp Fig 11B). Thresholded edges were binarized: ICC values above the 95th percentile were set as one and the rest set as zero. We then computed what proportion of the retained edges overlapped between the different age

windows using the Jaccard Similarity Index, ranging from 0 (no overlap) to 1 (perfect overlap). (Jaccard, 1912) A strong overlap, consistent across adjacent age groups, would indicate that edges contributing to either self- or others-identifiability were similar across individuals over the lifespan. We found that the overlap of edges contributing to the highest self-identifiability was low between age windows: less than 25% of edges within each network overlapped between age windows. The only exceptions were the visual and somatomotor networks, where the overlap was closer to 50% and 30%, respectively. In contrast, edges contributing the highest to others-identifiability across individuals were similar across different age windows across the lifespan: between 50% to 75% of edges within each network overlapped between different age windows. This was the case whether using the whole brain connectome, within- or between-network edges, and using different sliding window parameters (not shown).

We tested the extent edges contributing to self-identifiability also contributed to others-identifiability (Supp Fig 11C). Within each age window, we computed the Jaccard Similarity Index between the binarized ICC values for edges contributing to self-identifiability and the binarized ICC values for edges contributing to others-identifiability. Here, a strong overlap indicates that edges contributing to self-identifiability also contributed to others-identifiability. We found that, across age windows, edges contributing to self-identifiability showed little overlap (low Jaccard Index) with edges contributing to others-identifiability. This finding was consistent when using either whole brain connectome, within- or between-network edges, or when changing the window parameters (not shown).

Finally, we computed and plotted to which extent each node had a high number of edges contributing to self- (Supp Fig 12) or others-identifiability (Supp Fig 13). Following Amico & Goñi (2018), we computed the nodal density for each of the 400 nodes included in the Schaefer

atlas. For each node, we summed the number of edges above the 95th percentile contributing to either self- or others-identifiability and divided this number by the total number of edges per region (400). The resulting nodal density indicates to which extent edges from a given node contribute to self- or others-identifiability. Overall, consistent with our previous result, all nodes tended to contribute relatively similarly to self-identifiability.

c. **Clustering**

We additionally used a clustering approach to confirm that FC patterns across the whole brain at the individual-level differed from group-level FC. This was to confirm that there were no evident patterns of individual-level FC that were shared across individuals and age windows, which we would expect to see if specific edges contributed to self-identifiability. In each age window, we applied a 7-cluster K-means clustering approach to FC edges in two ways: on the average FC values of all edges at group-level and on the FC values of all edges for each participant separately. We then computed the overlap between the clusters obtained at the group-level and at the individual-level, with the reasoning that individual-level cluster assignments should overlap strongly with group-level assignments if FC patterns are more similar across individuals. In line with the other previous analyses, we found very little overlap between the clusters obtained at the group-level and the clusters obtained at the individual-level (average Jaccard score across cluster labels and windows of 0.06; Supp Fig 14). Furthermore, the overlap between individual- and group-level clusters varied between age windows, highlighting again that slightly different group selection yields different results.

Supplementary Figures

Supplementary Figure 1

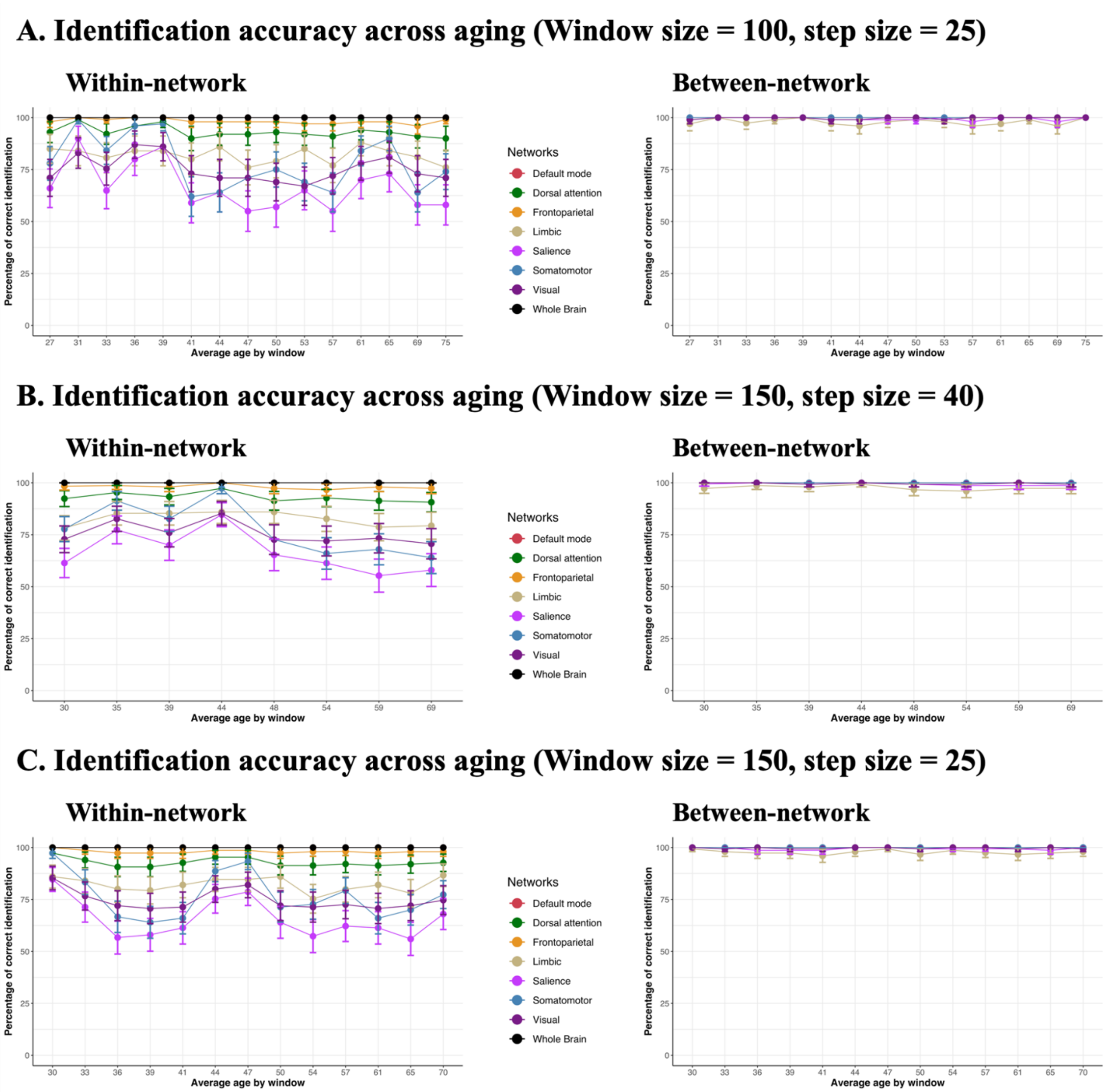

Supplementary Figure 1 - Fingerprint identification accuracy across the lifespan using different window parameters. Like in Figure 2B, we illustrate the fingerprint identification accuracy using a sliding window approach, varying the size of the window (100 or 150) and the step size (40 or 25). Figure 2B uses a window size of 100 and a step size of 40. Accuracy using within-network (left) and between-network (right) edges are represented.

Supplementary Figure 2

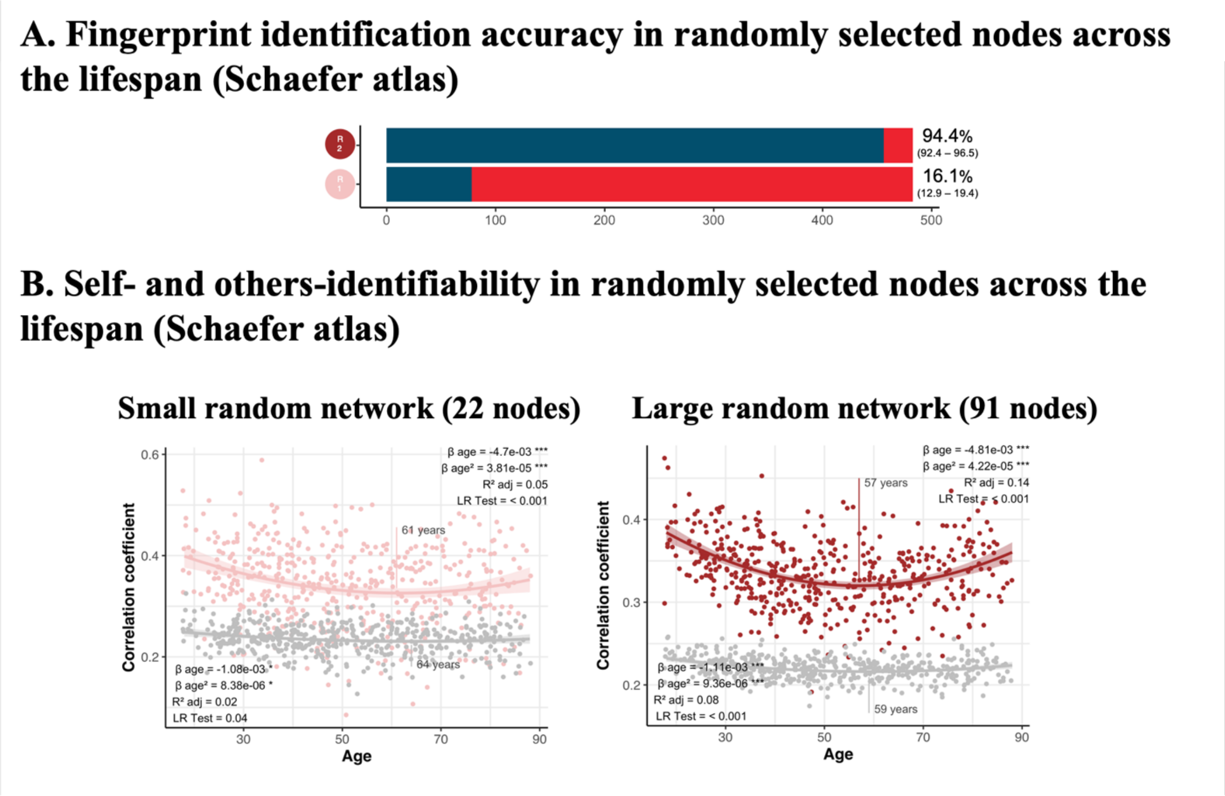

Supplementary Figure 2 – Fingerprint identification accuracy, and self- and others-identifiability using a randomized subset of nodes. We randomly selected two subsets of nodes from the Schaefer atlas across all regions and calculated the fingerprint identification accuracy (panel **A**) and the self- and others-identifiability (panel **B**) in both networks. The number of nodes were chosen to mirror the number of nodes in the smallest (limbic – 22 nodes) and the largest (default-mode – 91 nodes) Yeo networks using the Schaefer atlas. Edges within the randomly selected nodes were used to calculate the accuracy and the identifiability.

Supplementary Figure 3

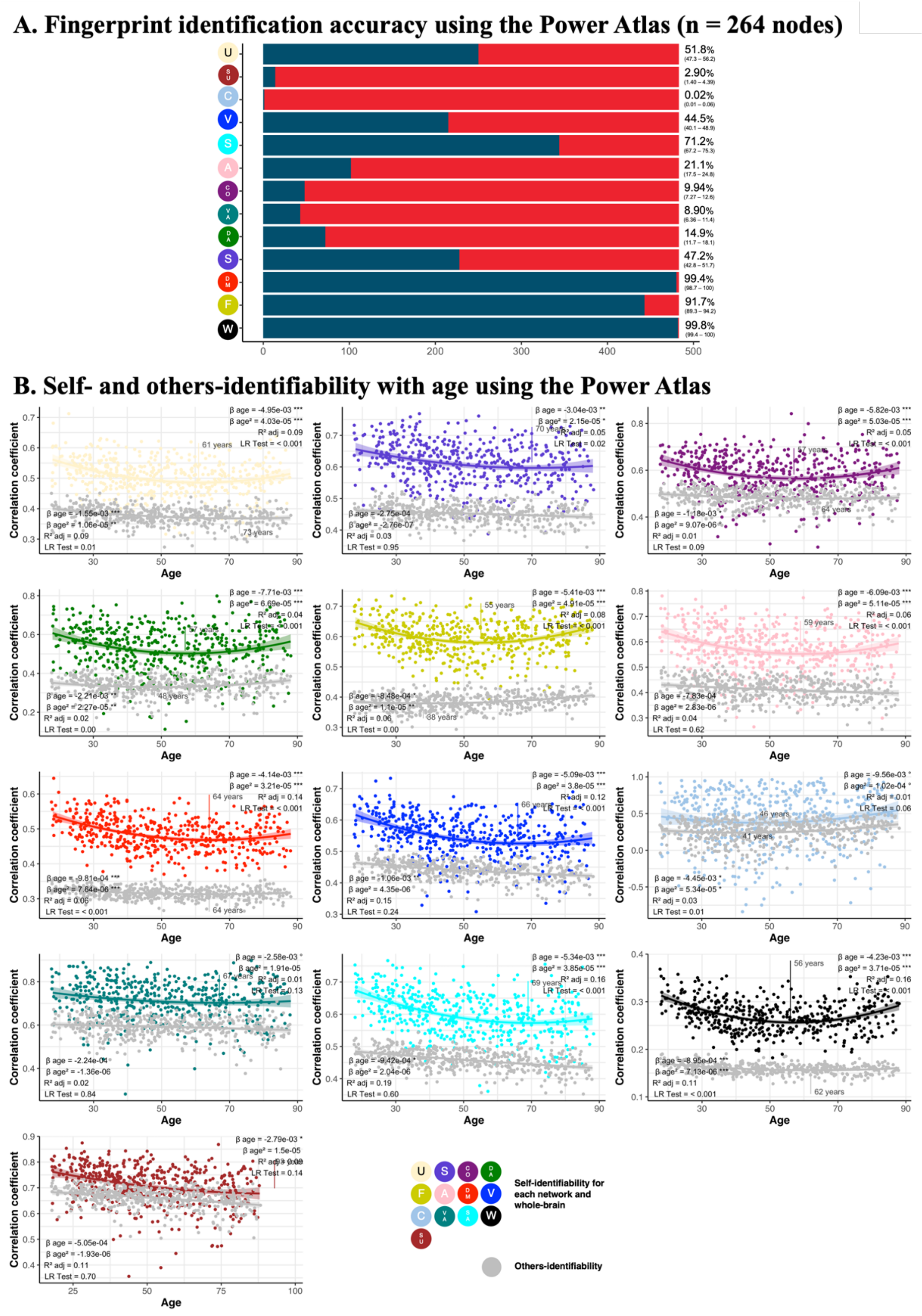

Supplementary Figure 3 – Fingerprint identification accuracy, and self- and others-identifiability using the Power atlas. We replicated our results from Figure 2A and Figure 3A using a different brain

parcellation: the Power atlas (Power et al. 2011). All networks from the Power atlas were replicated and the network acronyms are as follow: U (beige) = Uncertain, SA (light purple) = Salience, CO (dark purple) = Cingulo-opercular, DA (dark green) = Dorsal attention, F (light green) = Frontoparietal, A (pink) = Auditory, DM (red) = Default-mode, V (blue) = Visual, C (light blue) = Cerebellar, VA (teal) = Ventral attention, S (cyan) = Somatomotor, W (black) = Whole brain, SU (brown) = Subcortical.

Supplementary Figure 4

A. Within-network edges

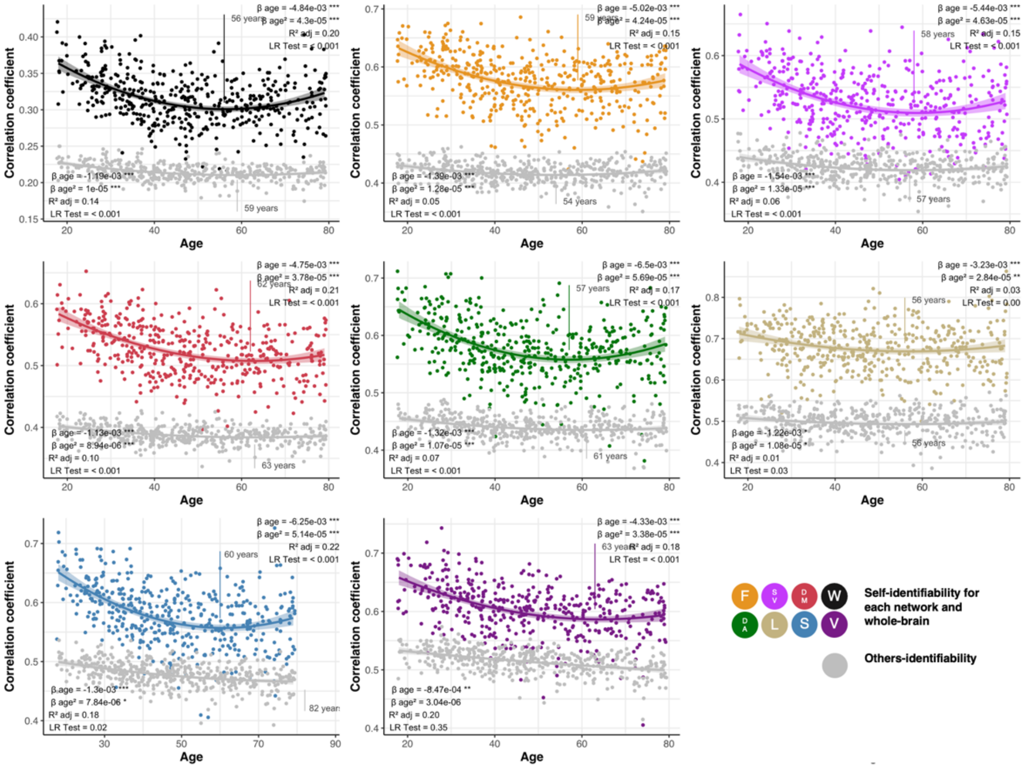

B. Between-network edges

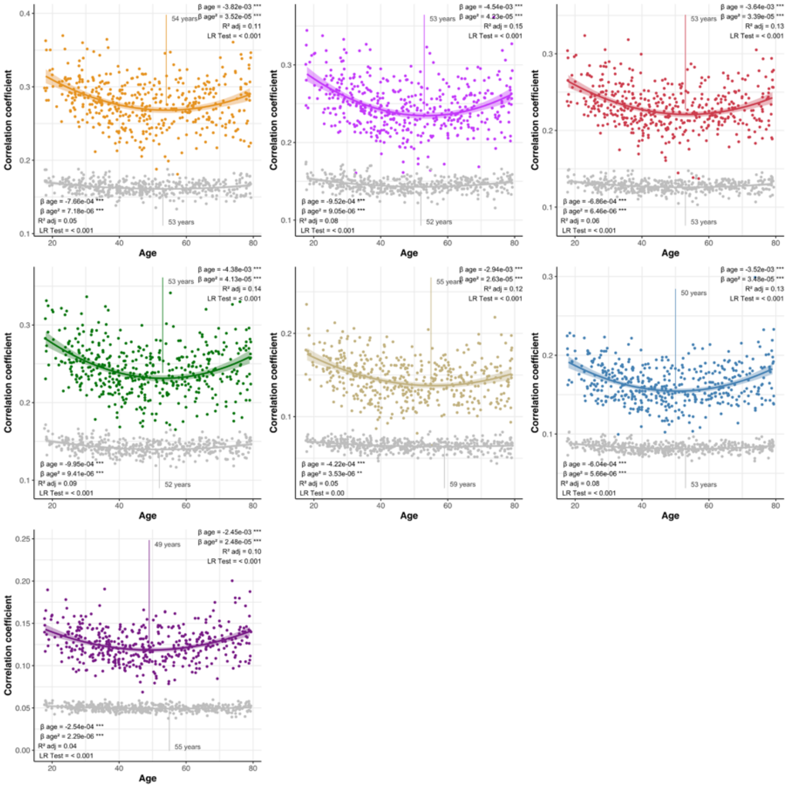

Supplementary Figure 4 – Differences in self- and others-identifiability across the lifespan, excluding the last decade. Change in self-identifiability (colors) and others-identifiability (grey) are represented using either within-network edges (A.) or between-network edges (B.). Each graph represents a different network, following acronyms and color schemes of Figure 1B. The beta coefficient of the age term and its quadratic term are presented at the top of the graph. We also present the adjusted R^2 of the model and the p-value of the nested likelihood ratio indicating the non-linearity of the relationship. The p-value of predictors surviving inclusion of covariates and execution of the bootstrapping are indicated by asterisks ($p < 0.001$ = ***, $p < 0.01$ = **, $p < 0.05$ = *). The age at which the curve changed direction was calculated from Stimson's equation and is illustrated on the graphs. Participants aged 80-89 were excluded.

Supplementary Figure 5

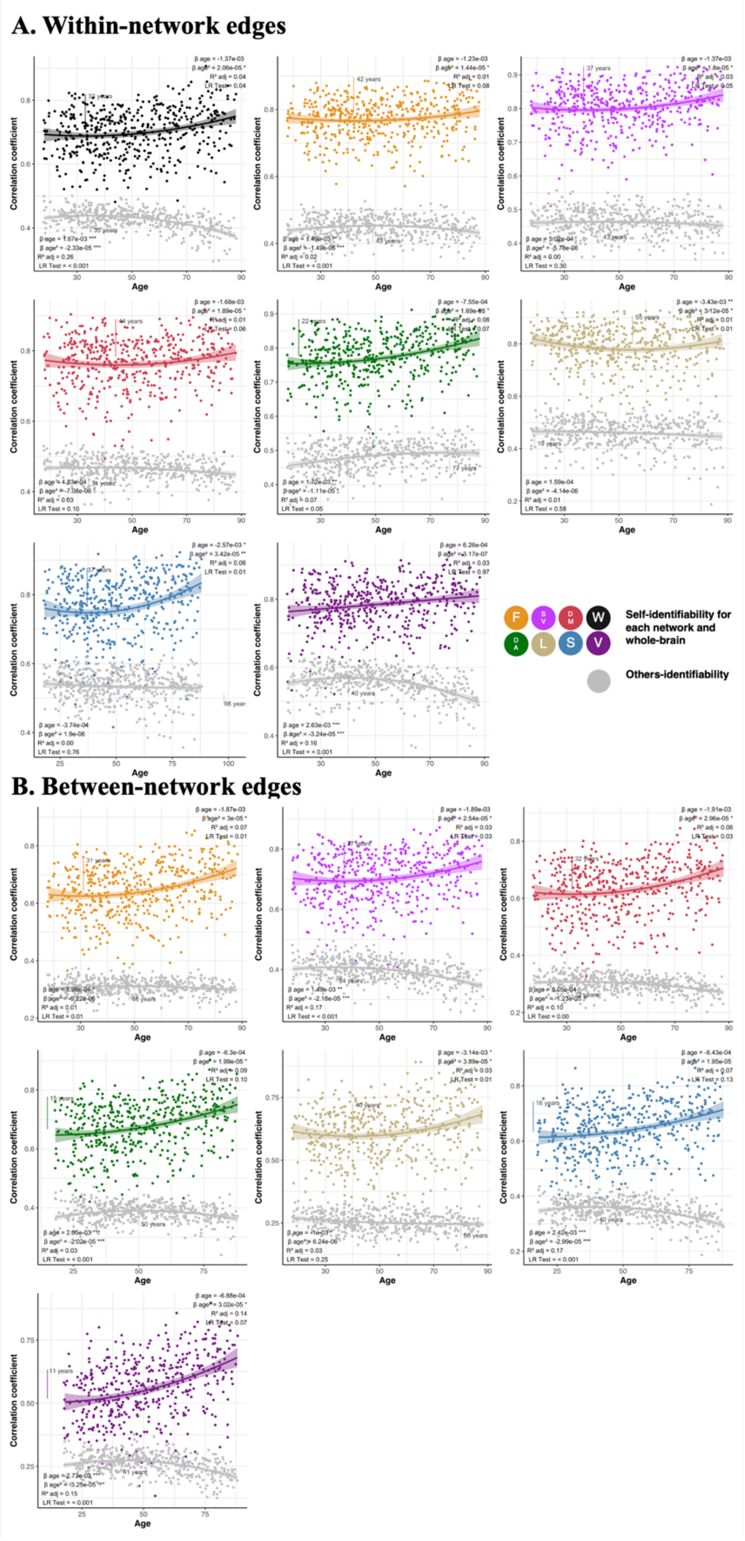

Supplementary figure 5 – Association between self- and others-identifiability and age across the lifespan using product–moment-calculated functional connectivity. Change in self-identifiability (colors) and others-identifiability (grey) are represented using either within-network edges (A.) or between-network edges (B.). Each graph represents a different network, following acronyms and color schemes of

Figure 1B. The beta coefficient of the age term and its quadratic term are presented at the top of the graph. We also present the adjusted R^2 of the model and the p-value of the nested likelihood ratio indicating the non-linearity of the relationship. The p-value of predictors surviving inclusion of covariates and execution of the bootstrapping are indicated by asterisks ($p < 0.001$ = ***, $p < 0.01$ = **, $p < 0.05$ = *). The age at which the curve changed direction was calculated from Stimson's equation and is illustrated on the graphs.

Supplementary figure 6

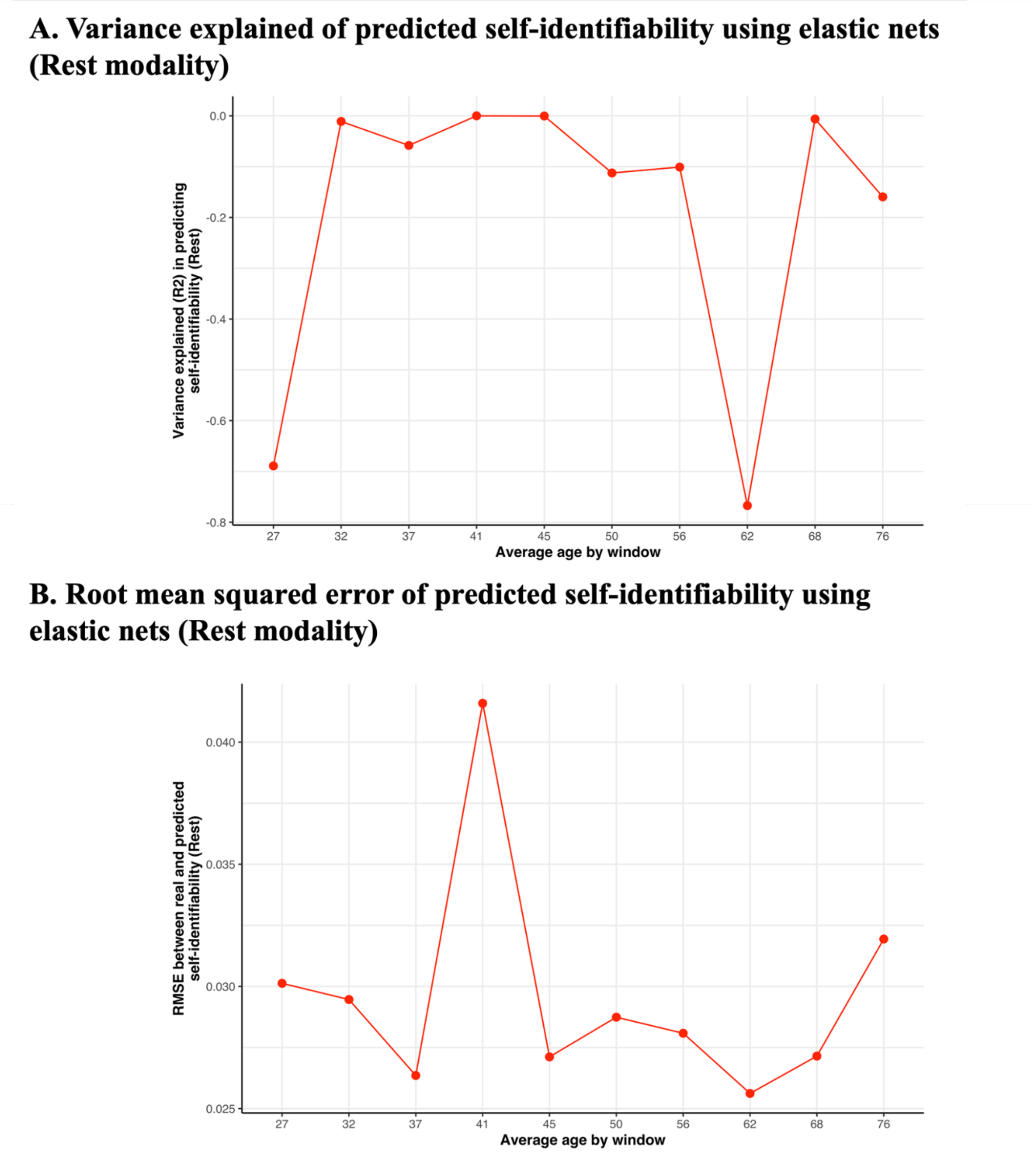

Supplementary Figure 6 – Model performance of elastic net models predicting self-identifiability from FC edges. Performance of the elastic net models is presented with two metrics: variance explained (R^2; A) and root mean square error (RMSE; B) for each age window using a window size of 100 and a step size of 40.

Supplementary Figure 7

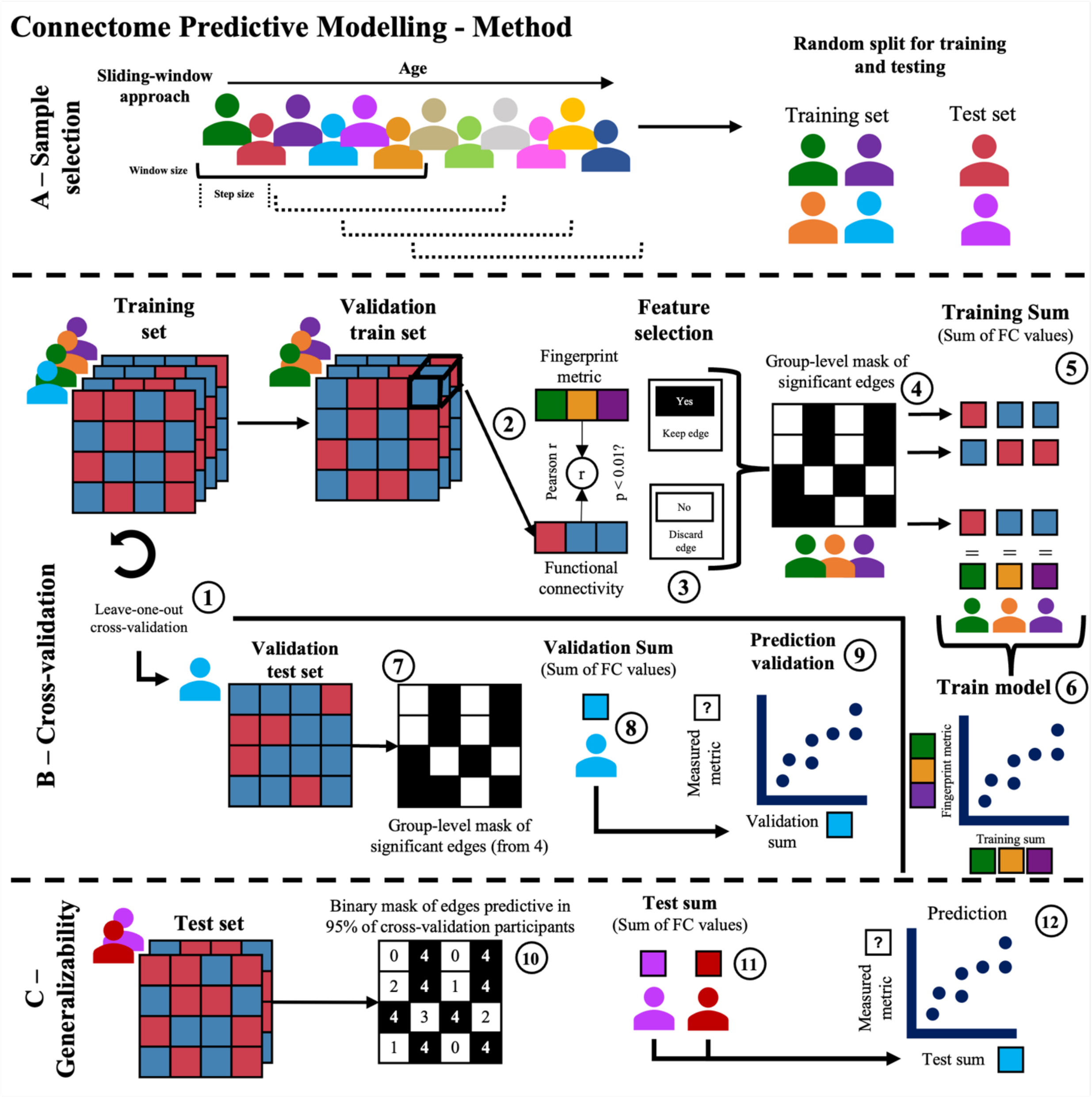

Supplementary Figure 7 - Illustration of the connectome predictive modelling

Illustration of the connectome predictive modelling in a simulated sample. The modelling is done in three main steps: Sample selection, Cross-validation, and Generalizability. Briefly, subsets of participants are chosen using a sliding-window approach. Then, functional connectivity in each edge is correlated to the fingerprint metric of interest during the cross-validation. Significantly correlated edges are used to form a mask in which we sum the FC values. These sums are then used to train a model predicting the fingerprint metric of interest. For each cross-validation, the mask derived in step 4 is applied to the left-out participant (step 7) and the sum of FC values of that participant is used to predict the fingerprint metric. In the final generalization step, edges predictive of the fingerprint metric in 95% of participants are kept. We then sum the FC values within these significant edges and use the sums to predict the fingerprint metric in the participants of the left-out test set (step 12). Both the self- and others-identifiability used in the modelling are calculated in the whole sample before the modelling.

Supplementary Figure 8

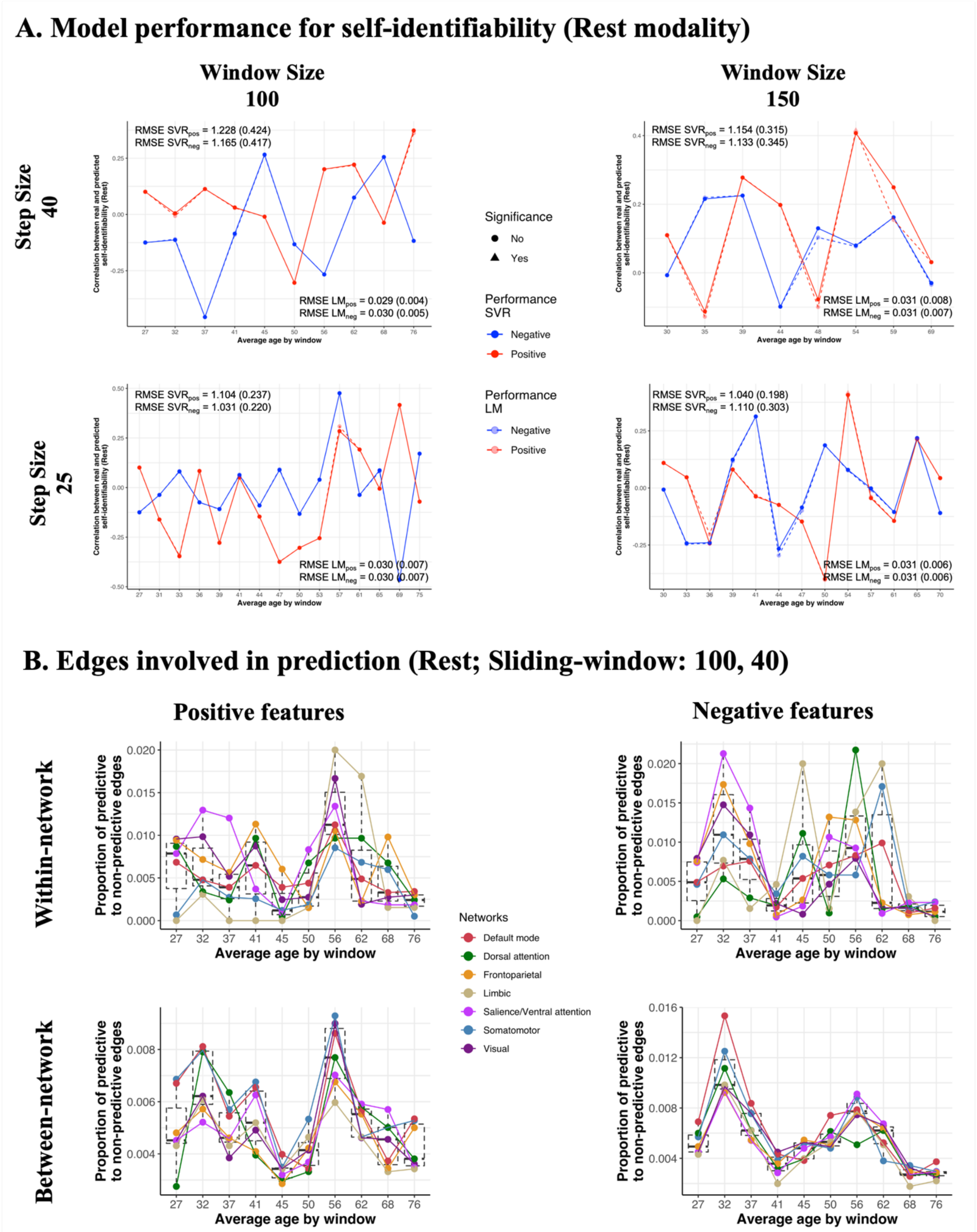

Supplementary Figure 8 – Connectome predictive modelling performance and predictive edges of self-identifiability (Rest modality). Point and line graphs representing **A.** model performance (correlation of predicted and measured fingerprint values in the test set) and **B**. network over-representation of predictive edges in predicting self-identifiability. In **A.**, models using support vector regression (SVR) are

presented in dashed lines and models using linear models (LM) are presented in solid lines. Average root mean square error (RMSE) across windows is presented in each graph. Graphs are presented by window size (number of participants) and step size (difference in participants included compared to previous windows). In **B.**, representative example of the proportion of edges predictive of self-identifiability in each network following cross-validation. The proportion is the number of predictive edges over the total size of the network.

Supplementary Figure 9

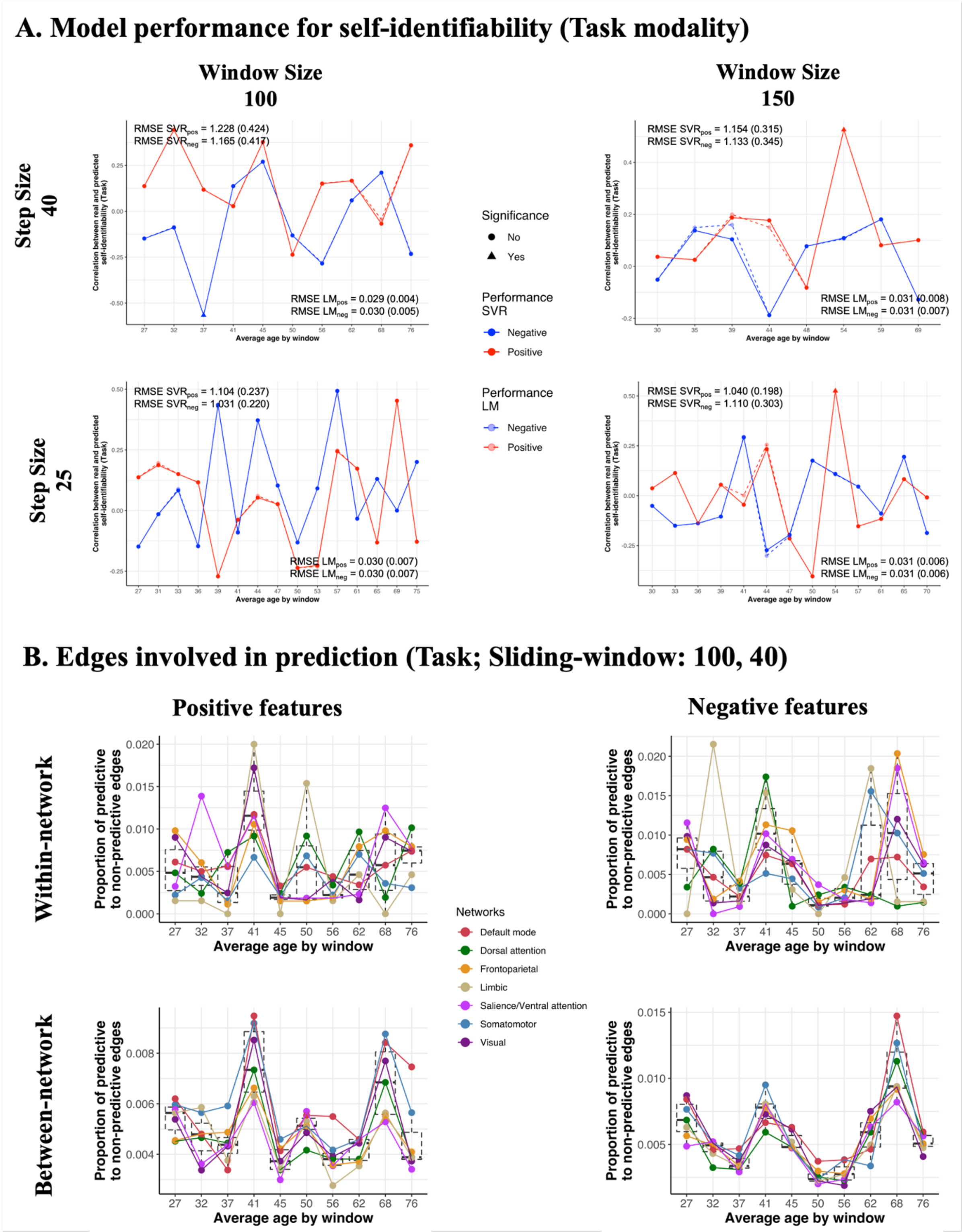

Supplementary Figure 9 - Connectome predictive modelling performance and predictive edges of self-identifiability (Task modality). Point and line graphs representing **A.** model performance (correlation of predicted and measured fingerprint values in the test set) and **B**. network over-representation of predictive edges in predicting self-identifiability. In **A**., models using support vector regression (SVR) are

presented in dashed lines and models using linear models (LM) are presented in solid lines. Average root mean square error (RMSE) across windows is presented in each graph. Graphs are presented by window size (number of participants) and step size (difference in participants included compared to previous windows). In **B.**, representative example of the proportion of edges predictive of self-identifiability in each network following cross-validation. The proportion is the number of predictive edges over the total size of the network.

Supplementary Figure 10

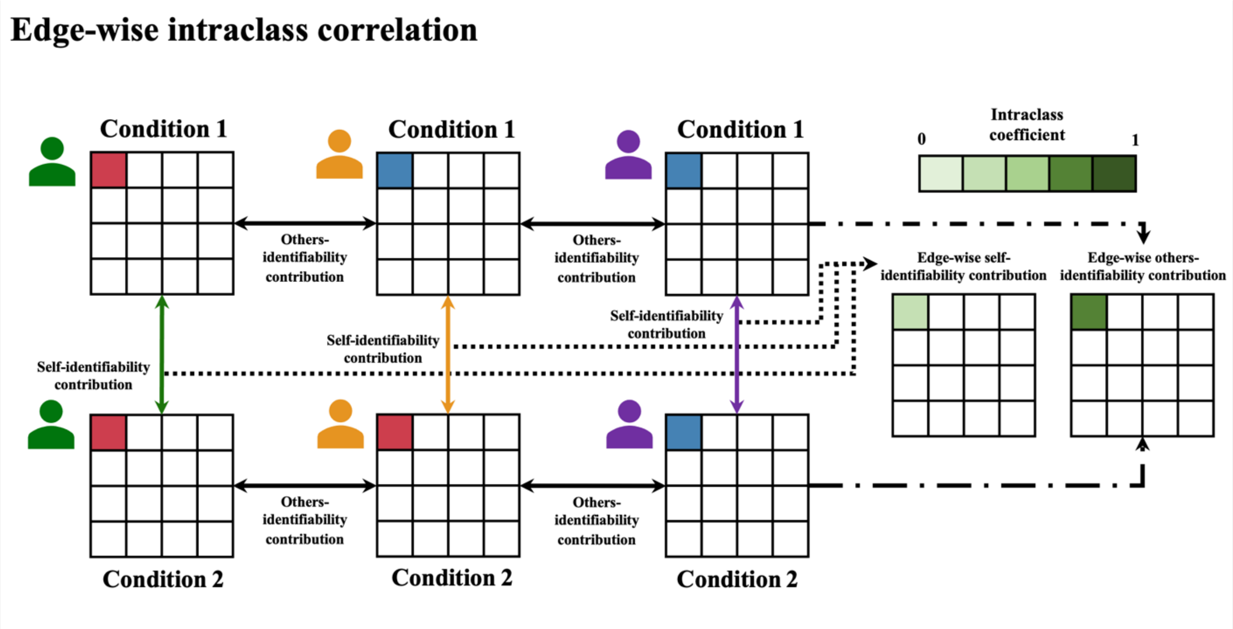

Supplementary Figure 10 - Illustration of the edge-wise intra-class correlation (ICC) methodology adapted from Amico & Goñi (2018). For each edge, we derive that edge's contribution to self-identifiability and to others-identifiability (ICC coefficient ranging from 0 to 1). A high ICC for self-identifiability indicates that the edge's contribution to self-identifiability is shared across individuals. In contrast, a high ICC for others-identifiability indicates that the edge's contribution to others-identifiability is shared across individuals.

Supplementary Figure 11

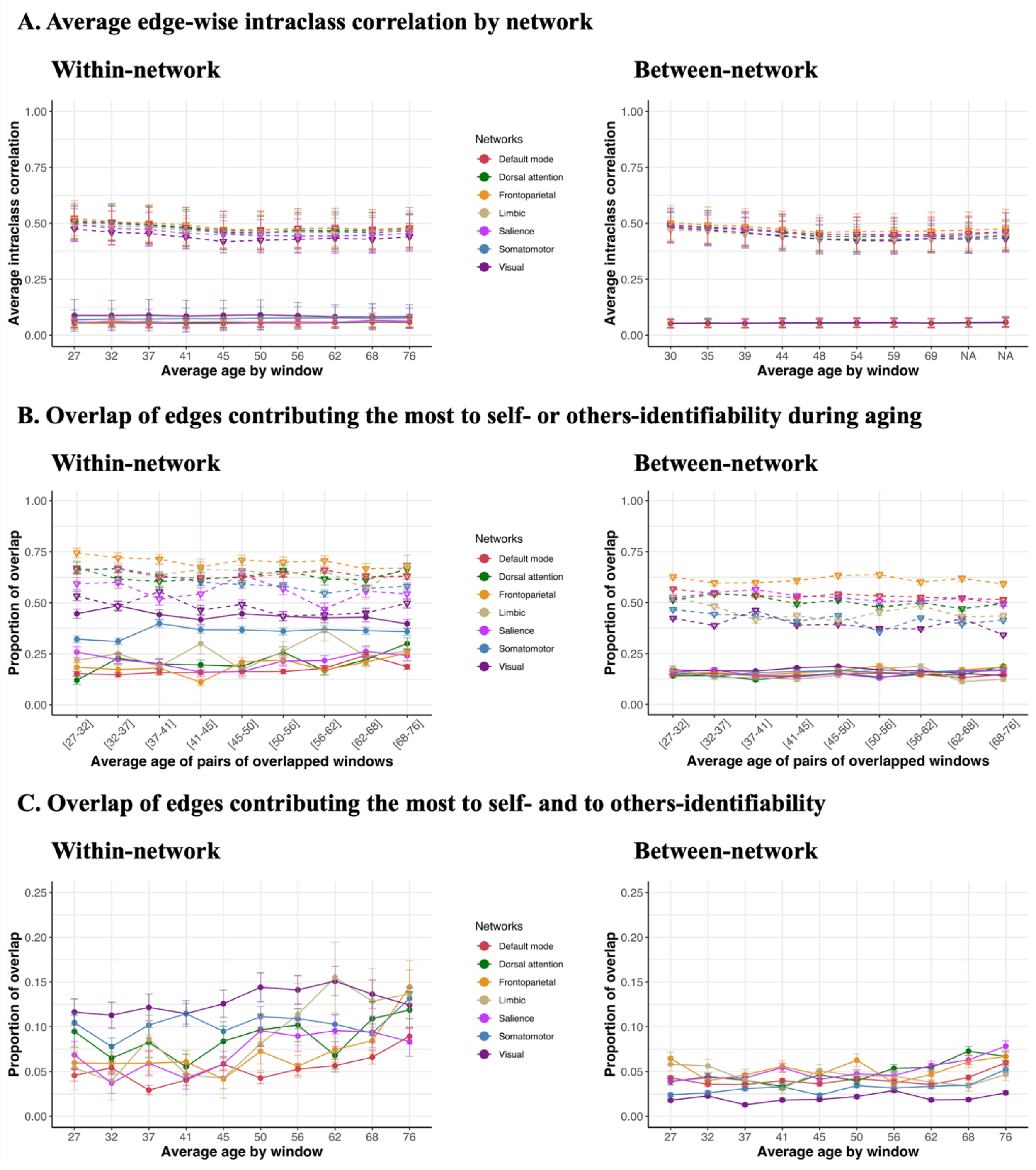

Supplementary Figure 11 - Edge-wise contribution to self- and others-identifiability (window size = 100, step size = 40) A. Average intraclass correlation (ICC) coefficients by network—for within- and between-network edges—in edges contributing the highest to self- and others-identifiability (values within each age window above the 95th percentile). Full circles and lines represent the average ICC of edges contributing the highest to self-identifiability while downward facing triangles and dashed lines represent the average ICC of edges contributing the highest to others-identifiability. Each average is accompanied by the standard deviation as an error bar. **B.** Overlap of edges contributing the most to self- (full lines and circles) and to others-identifiability (dashed lines and triangles), as measured by the Jaccard Similarity

Index (0 = no overlap, 1 = perfect overlap). The average age of age windows demonstrating overlap are indicated in square brackets. The 95% confidence interval of the standard error for the proportion of overlap, as a function of each network size, is illustrated with error bars. **C.** Overlap of edges contributing the most to both self- and others-identifiability within each age window as measured by the Jaccard Similarity Index. The 95% confidence interval of the standard error for the proportion of overlap is illustrated with error bars.

Supplementary Figure 12

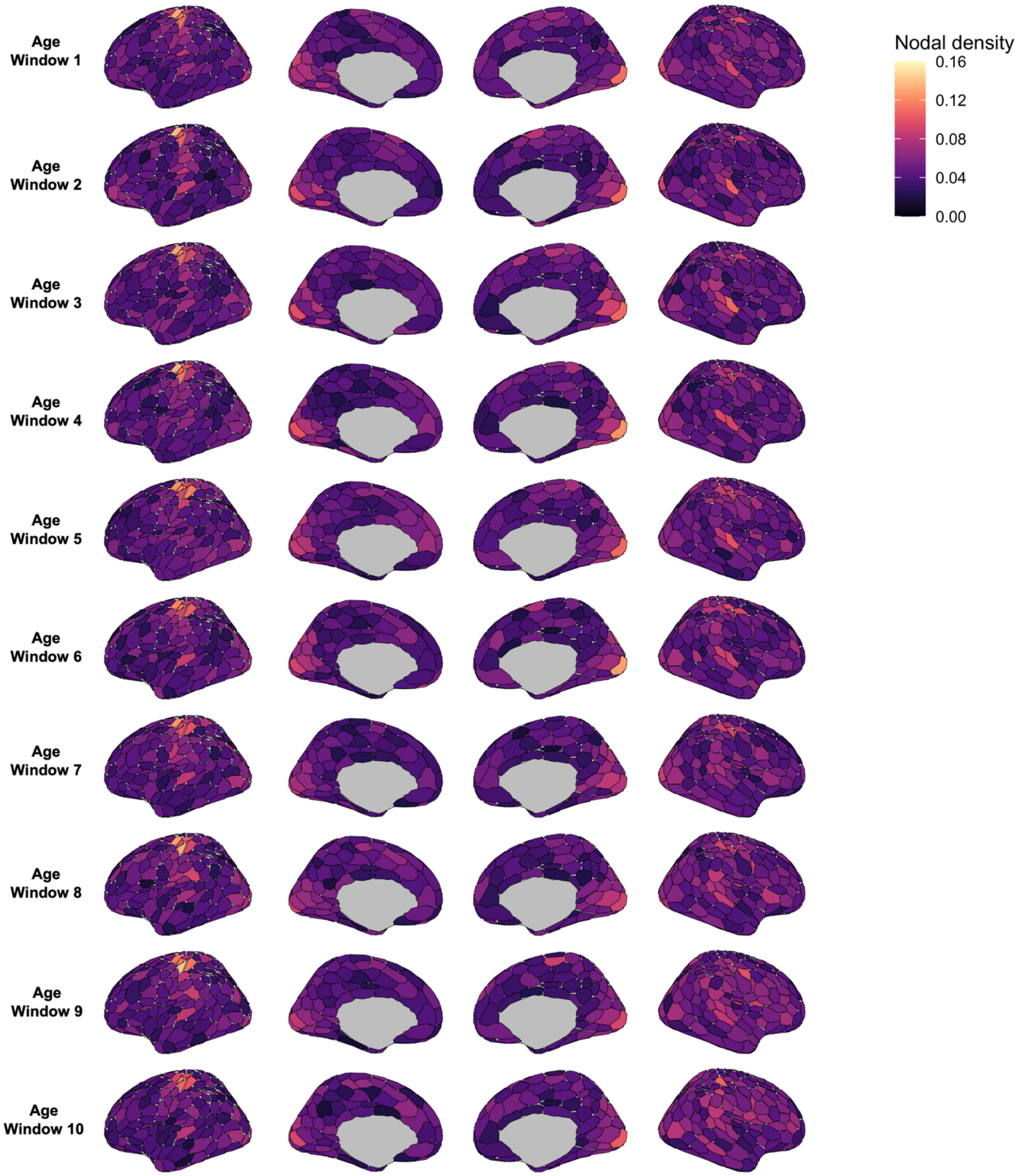

Supplementary Figure 12 – Nodal density of edges highly contributing to self-identifiability. For each age window, we plot the nodal density (sum of number of edges above the 95th percentile threshold of the highest intraclass correlation divided by the number of edges for a given node) using the Schaefer atlas (400 nodes). A higher nodal density means that the node had a higher proportion of edges contributing to self-identifiability.

Supplementary Figure 13

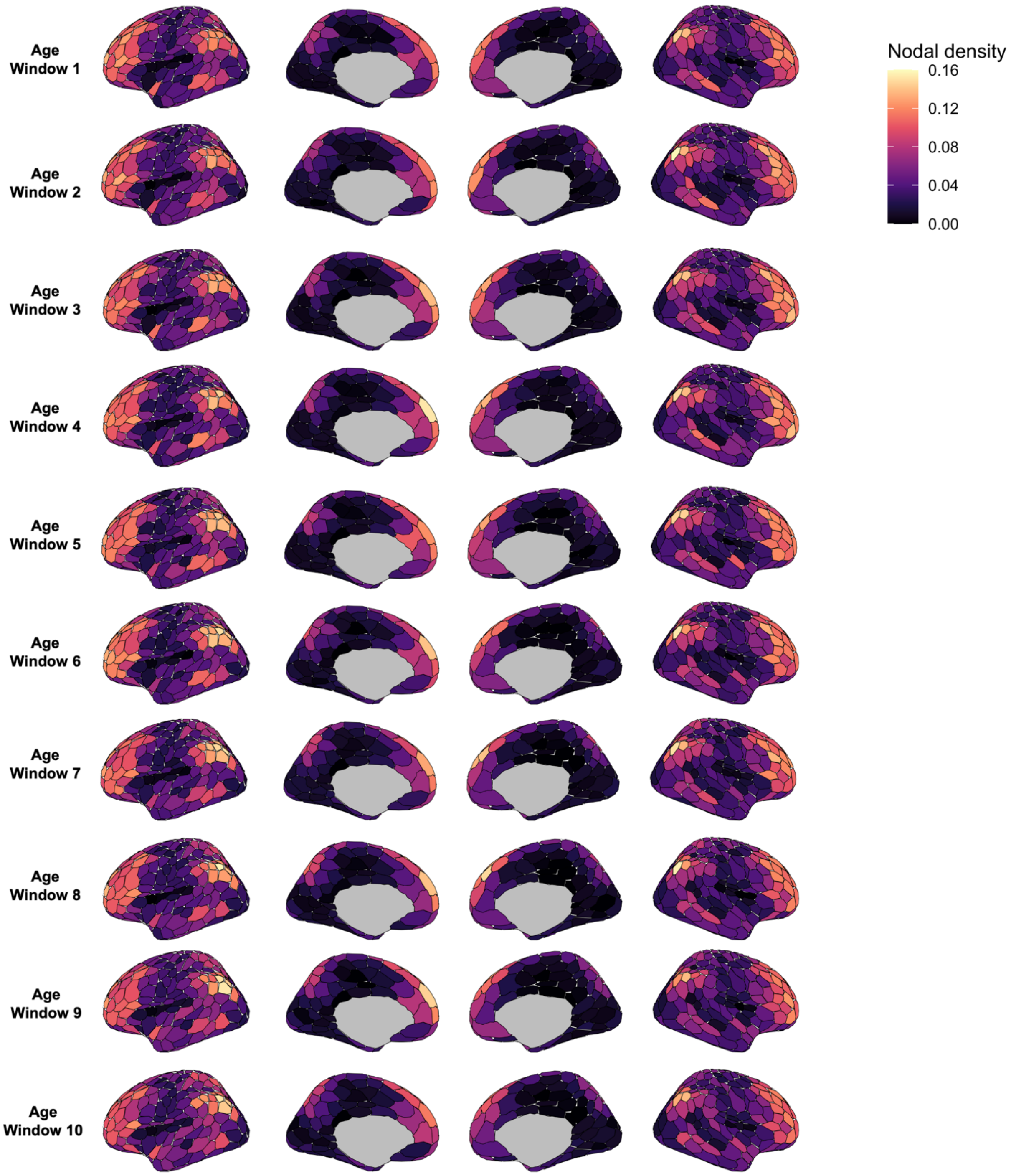

Supplementary Figure 13 – Nodal density of edges highly contributing to others-identifiability. For each age window, we plot the nodal density (sum of edges above the 95th percentile threshold of the highest intraclass correlation divided by the number of edges for a given node) using the Schaefer atlas (400 nodes). A higher nodal density means that the node had a higher proportion of edges contributing to others-identifiability.

Supplementary Figure 14

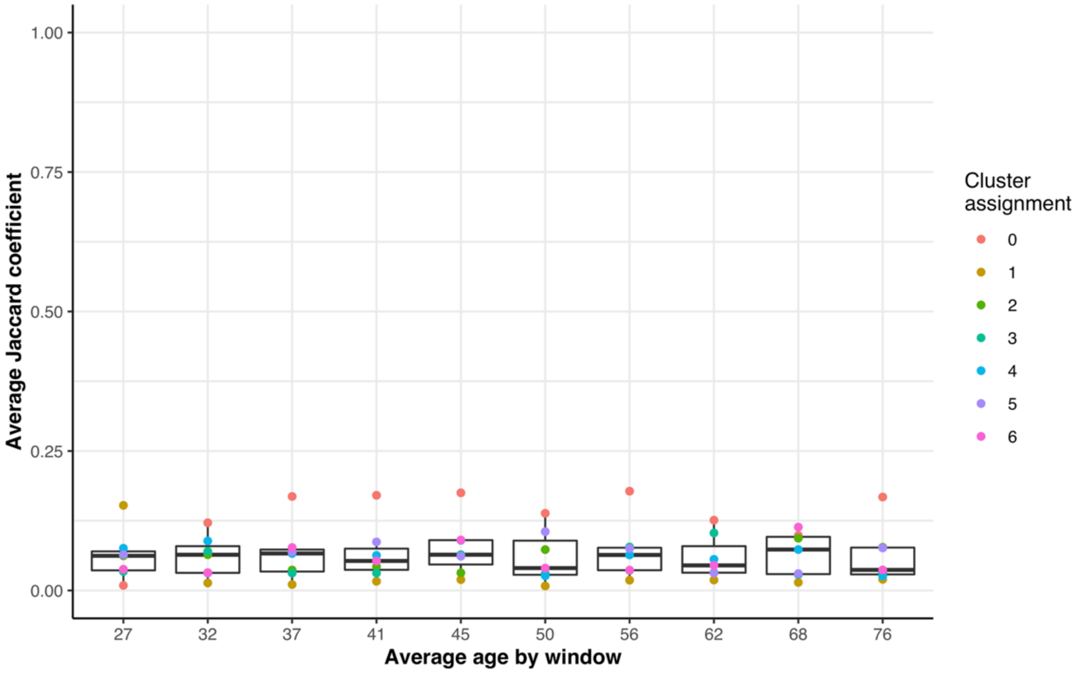

Supplementary Figure 14 – Average overlap between group-level and individual-level clustering of functional connectivity edges. For each cluster label derived by the 7-cluster K-means, we computed the average overlap between the cluster labels assigned to group-level connectivity and the individual-level connectivity using the Jaccard coefficient. A coefficient closer to 1 indicates that the clusters overlap more between group- and individual-level connectivity, while a coefficient closer to 0 indicates that the clusters overlap less between group- and individual-level connectivity.

Supplementary Figure 15

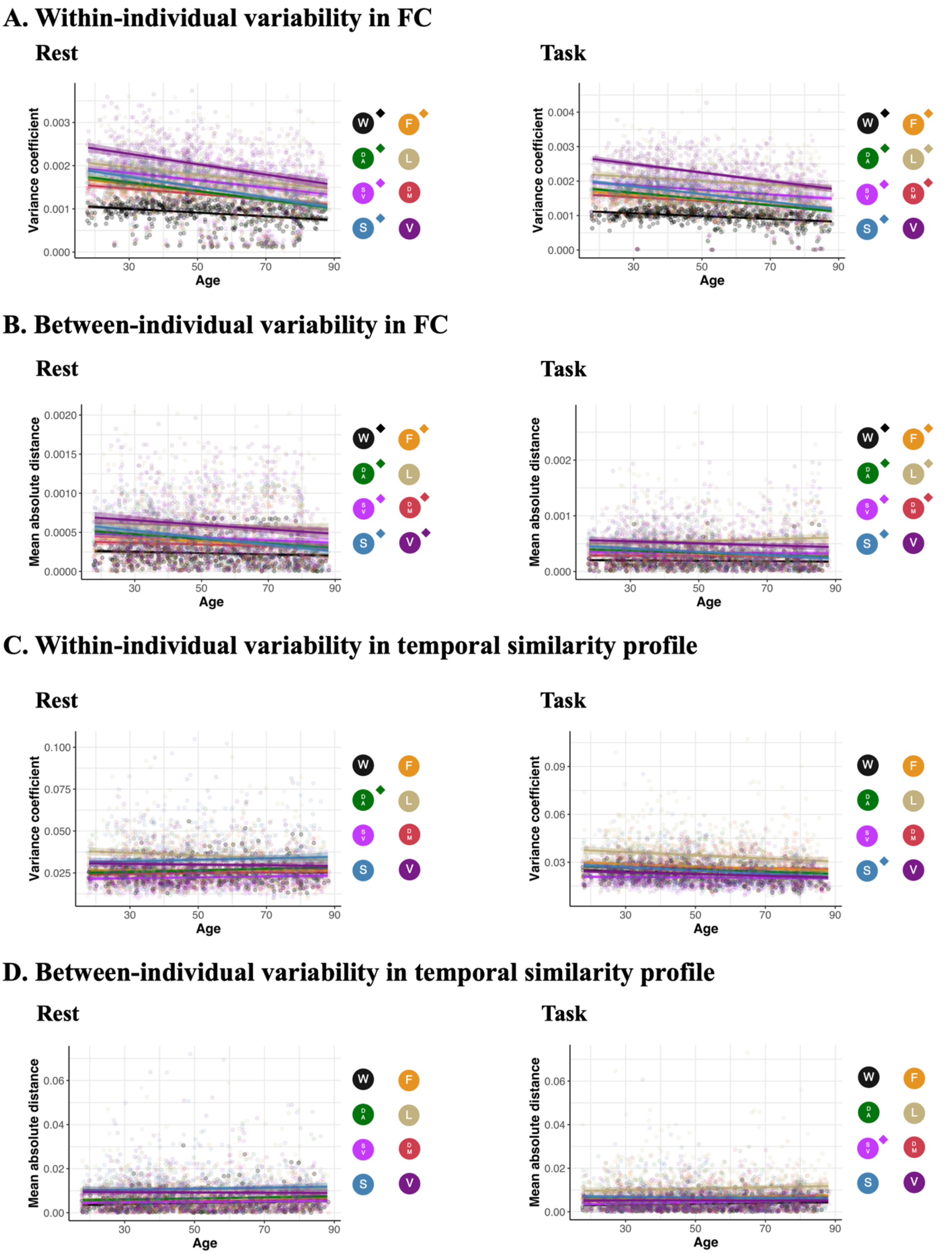

Supplementary Figure 15 - Change in within- and between-individual variability in functional connectivity across the lifespan and across tasks. This figure presents the association between FC variability within- (panel **A.**) and between-individual (panel **B.**) and age. It also presents the association between variability in temporal similarity profile within- (panel **C.**) and between-individuals (panel **D.**). Each network is color-coded based on the abbreviation and color scheme presented in Figure 1B. A lozenge next to the network acronym representing the network at the bottom of each graph indicates that the model survived controlling for confounders and bootstrap resampling.

Supplementary figure 16

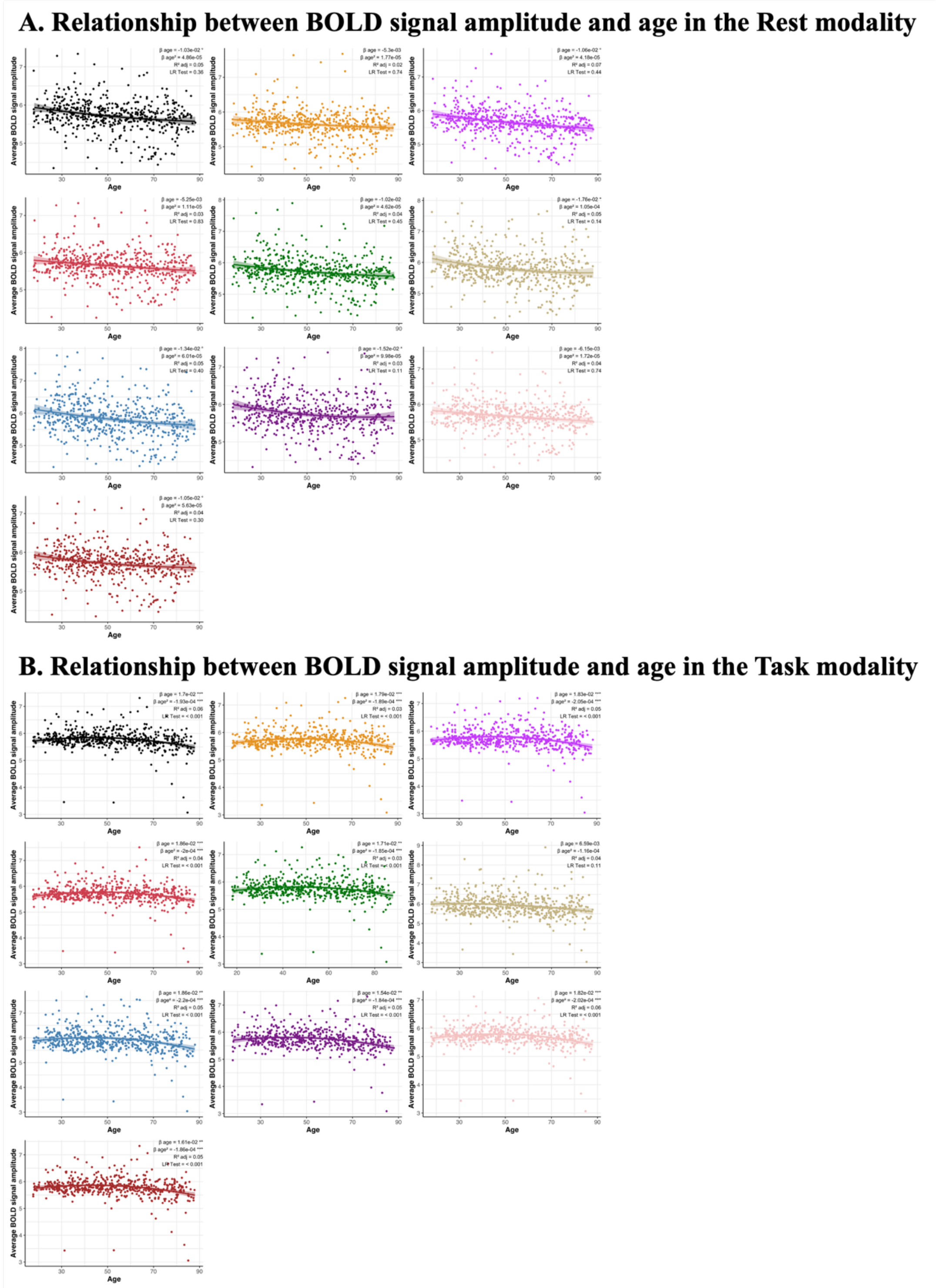

Supplementary Figure 16 – Association between BOLD signal amplitude and age across the lifespan. The amplitude of the fMRI BOLD signal was computed in both the Rest (A) and Task (B) modality separately. Then, the amplitude was related to age using a non-linear (quadratic) model. The beta coefficient of the age term and its quadratic term are presented at the top of the graph. We also present the adjusted R^2 of the model and the p-value of the nested likelihood ratio indicating the

non-linearity of the relationship. The p-value of predictors surviving inclusion of covariates and execution of the bootstrapping are indicated by asterisks ($p < 0.001$ = ***, $p < 0.01$ = **, $p < 0.05$ = *, $p < 0.1$ = °).

Supplementary Figure 17

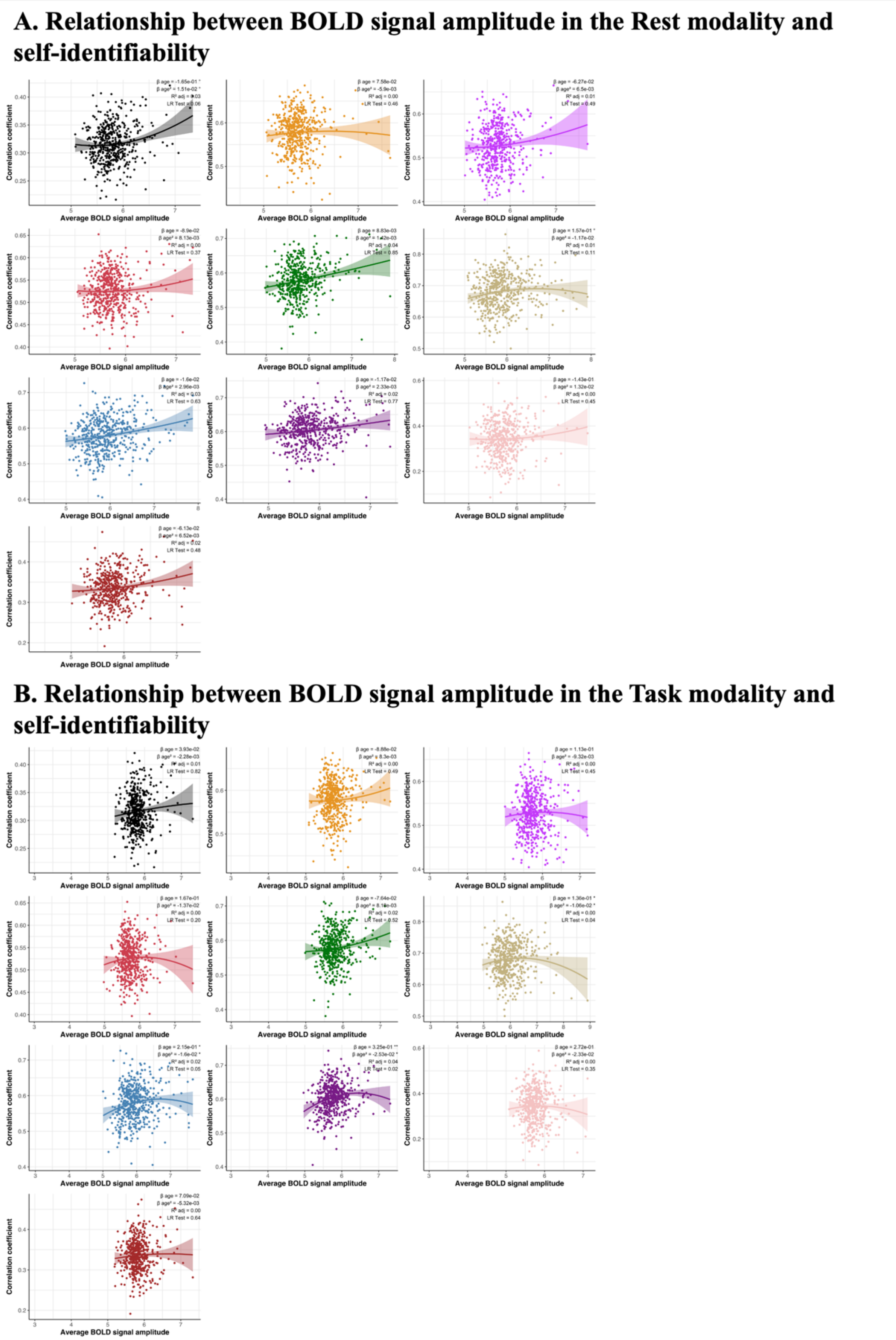

Supplementary Figure 17 – Association between BOLD signal amplitude and self-identifiability across the lifespan. The average amplitude of the fMRI BOLD signal was computed in both the Rest (A) and Task (B) modality separately. Then, the amplitude was related to age using a non-linear (quadratic)

model. The beta coefficient of the age term and its quadratic term are presented at the top of the graph. We also present the adjusted R^2 of the model and the p-value of the nested likelihood ratio indicating the non-linearity of the relationship. The p-value of predictors surviving inclusion of covariates and execution of the bootstrapping are indicated by asterisks ($p < 0.001$ = ***, $p < 0.01$ = **, $p < 0.05$ = *, $p < 0.1$ = °).

Supplementary Figure 18

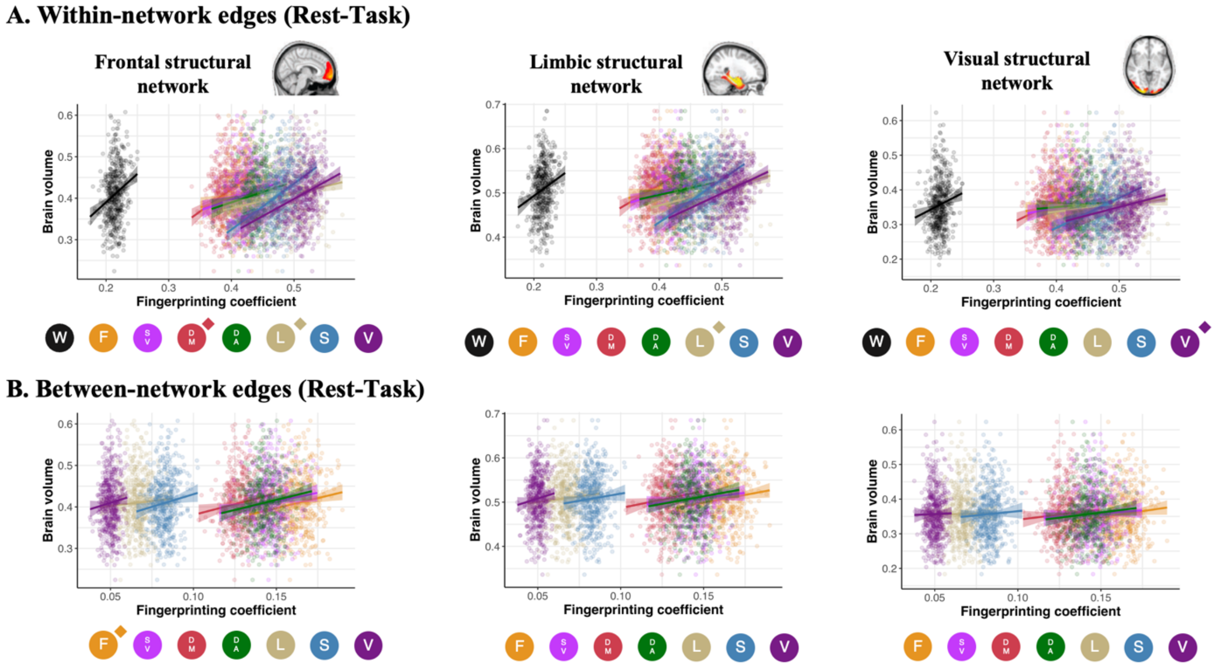

Supplementary Figure 18 - Association between grey matter volume and others-identifiability. Scatterplots presenting the association between others-identifiability and grey matter volume in three morphometric networks: frontal structural network (age-sensitive network), limbic structural network (Alzheimer's/age-related network) and visual structural network ("control" network). Data points, regression slopes and bubbles below the graph follow the color scheme of Figure 1B. A "◆" at the upper right of the bubble indicates that the association survived when using bootstrapping and controlling for covariates (including age). Panel **A.** presents the results for within-network edges while panel **B.** presents the results for the between-network edges.

Supplementary Figure 19

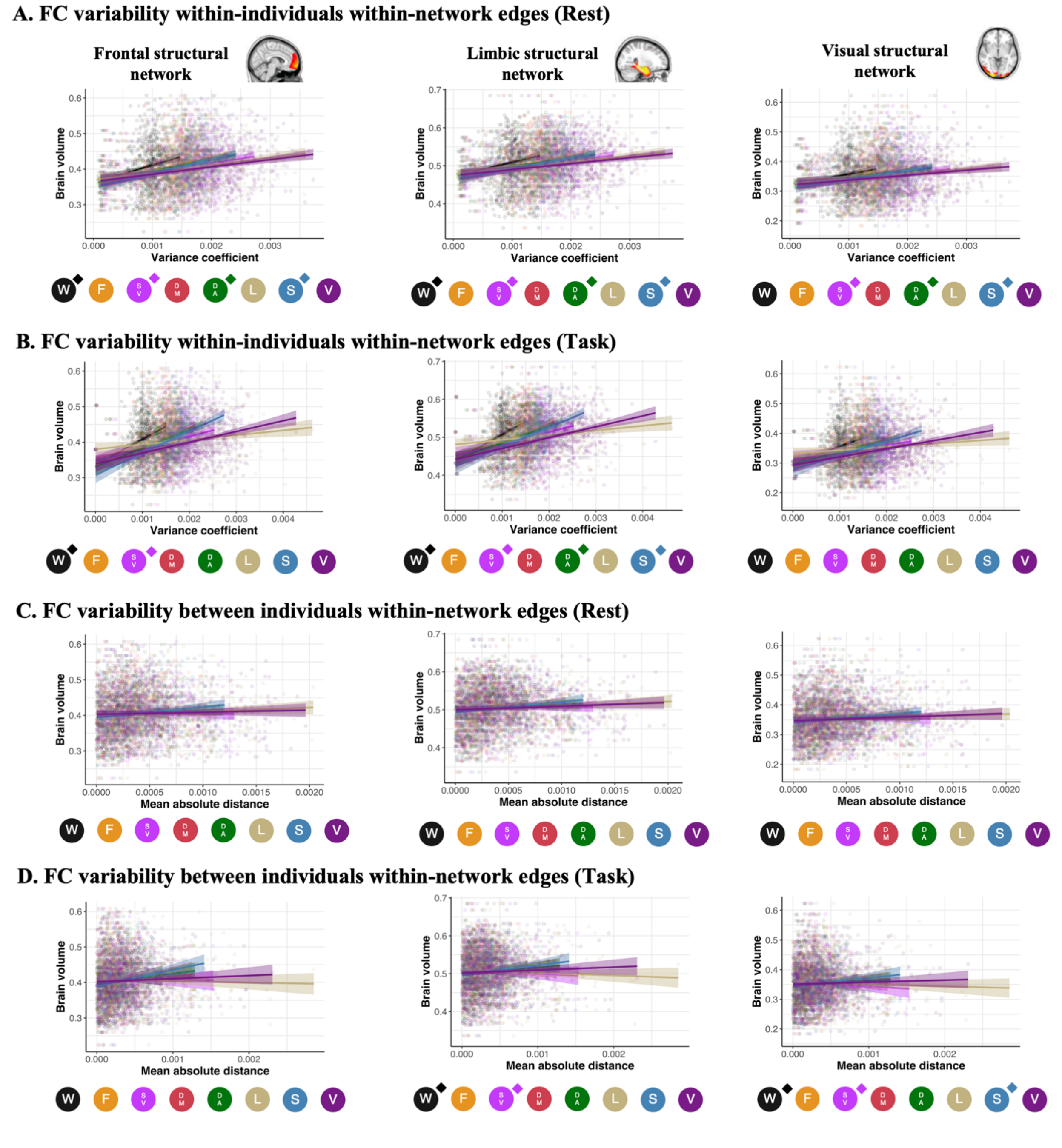

Supplementary Figure 19 – FC variability and grey matter associations. Scatterplots presenting the association between within-individual (panels **A.** and **B.**) and between-individual variability in FC (panels **C.** and **D.**), and grey matter volume in three morphometric networks: frontal structural network (age-sensitive network), limbic structural network (Alzheimer's/age-related network) and visual structural network ("control" network). Data points, regression slopes and bubbles below the graph follow the color scheme of Figure 1B. A "◆" at the upper right of the bubble indicates that the association survived when using bootstrapping and controlling for covariates (including age).

Supplementary Table 1 – McNemar tests comparing fingerprint identification accuracy obtained from within-network edges and between-network edges

Network	Within-network accuracy	Between-network accuracy	Statistic
**Visual**	75.6 (71.7-79.4)	99.8 (99.4-100)	χ^2 (df = 1, $p < 0.001$) = 113.08
**Somatomotor**	80.7 (77.2-84.3)	100 (-)	n/a
**Limbic**	81.2 (77.7-84.6)	97.9 (96.7-99.2)	χ^2 (df = 1, $p < 0.001$) = 63.92
**Dorsal attention**	93.2 (90.9-95.4)	100 (-)	n/a
**Default mode**	100 (-)	100 (-)	n/a
**Salience**	69.6 (65.6-73.7)	99.6 (99.0-100)	χ^2 (df = 1, $p < 0.001$) = 143.01
**Frontoparietal**	98.3 (97.2-99.5)	100 (-)	n/a

In cases where all participants were identified accurately, the McNemar test cannot be calculated.

Supplementary Table 2 – McNemar tests comparing the fingerprint identification accuracy between networks (within-network edges)

	**Visual (75.6%)**	**Somato-motor (80.7%)**	**Limbic (81.2%)**	**Dorsal attention (93.2%)**	**Default mode (100%)**	**Salience (69.6%)**	**Fronto-parietal (98.3%)**
**Visual (75.6%)**		χ^2 (df = 1, p = 0.037) = 4.331	χ^2 (df = 1, p = 0.012) = 6.322	χ^2 (df = 1, p < 0.001) = 62.284	n/a	χ^2 (df = 1, p = 0.0293) = 4.752	χ^2 (df = 1, p < 0.001) = 104.22
**Somato-motor (80.7%)**			χ^2 (df = 1, p = 0.603) = 0.271	χ^2 (df = 1, p < 0.001) = 44.444	n/a	χ^2 (df = 1, p < 0.001) = 23.025	χ^2 (df = 1, p < 0.001) = 79.281
**Limbic (81.2%)**				χ^2 (df = 1, p < 0.001) = 30.533	n/a	χ^2 (df = 1, p < 0.001) = 23.841	χ^2 (df = 1, p < 0.001) = 65.878
**Dorsal attention (93.2%)**					n/a	χ^2 (df = 1, p < 0.001) = 97.714	χ^2 (df = 1, p < 0.001) = 17.633
**Default mode (100%)**						n/a	n/a
**Salience (69.6%)**							χ^2 (df = 1, p < 0.001) = 133.17
**Fronto-parietal (98.3%)**							

McNemar tests were calculated for every pair of networks that didn't have perfect identification accuracy. To simplify the table, only the upper half was completed (the diagonal yields a chi-square of 0, and the lower half would be exactly symmetric to the upper half)

Supplementary Table 3 – McNemar tests comparing fingerprint identification accuracy from product–moment-derived FC and from partial correlation derived FC (within-network edges)

Network	Product–moment FC	Partial correlation FC	Statistic
**Visual**	76.6<br>(72.8-80.4)	75.6<br>(71.7-79.4)	χ^2 (df = 1, p = 0.705) = 0.144
**Somatomotor**	77.7<br>(73.9-81.4)	80.7<br>(77.2-84.3)	χ^2 (df = 1, p = 0.301) = 1.070
**Limbic**	79.3<br>(75.7-82.9)	81.2<br>(77.7-84.6)	χ^2 (df = 1, p = 0.423) = 0.643
**Dorsal attention**	91.9<br>(89.5-94.4)	93.2<br>(90.9-95.4)	χ^2 (df = 1, p = 0.450) = 0.571
**Default mode**	96.3<br>(94.6-98.0)	100<br>(-)	n/a
**Salience**	97.7<br>(96.4-99.1)	69.6<br>(65.6-73.7)	χ^2 (df = 1, p < 0.001) = 123.14
**Frontoparietal**	98.3<br>(97.2-99.5)	98.3<br>(97.2-99.5)	χ^2 (df = 1, p = 1) = 0
**Whole brain**	95.4<br>(93.6-97.3)	100<br>(-)	n/a

In cases where all the sample was identified accurately, the McNemar test cannot be calculated.

Supplementary Table 4 – McNemar tests comparing fingerprint identification accuracy from product–moment-derived FC and from partial correlation derived FC (between-network edges)

Network	Product–moment FC	Partial correlation FC	Statistic
**Visual**	66.7 (62.5-70.9)	99.8 (99.4-100)	χ^2 (df = 1, $p < 0.001$) = 158.01
**Somatomotor**	80.3 (76.8-83.8)	100 (-)	n/a
**Limbic**	90.0 (87.4-92.7)	97.9 (96.7-99.2)	χ^2 (df = 1, $p < 0.001$) = 24.45
**Dorsal attention**	93.2 (90.9-95.4)	100 (-)	n/a
**Default mode**	91.1 (88.6-93.6)	100 (-)	n/a
**Salience**	92.5 (90.2-94.9)	99.6 (99.0-100)	χ^2 (df = 1, $p < 0.001$) = 28.658
**Frontoparietal**	94.9 (93.1-97.0)	100 (-)	n/a

In cases where all the sample was identified accurately, the McNemar test cannot be calculated.

Supplementary references

Jaccard, P. (1912). THE DISTRIBUTION OF THE FLORA IN THE ALPINE ZONE. *New Phytologist*, *11*(2), 37–50. https://doi.org/10.1111/j.1469-8137.1912.tb05611.x

Koo, T., & Li, M. (2016). A Guideline of Selecting and Reporting Intraclass Correlation Coefficients for Reliability Research. *J Chiropr Med*, *15*(2), 155–163.

Scheinost, D., Noble, S., Horien, C., Greene, A. S., Lake, E. M., Salehi, M., Gao, S., Shen, X., O'Connor, D., Barron, D. S., Yip, S. W., Rosenberg, M. D., & Constable, R. T. (2019). Ten simple rules for predictive modeling of individual differences in neuroimaging. *NeuroImage*, *193*(March), 35–45. https://doi.org/10.1016/j.neuroimage.2019.02.057

Shen, X., Finn, E. S., Scheinost, D., Rosenberg, M. D., Chun, M. M., Papademetris, X., & Constable, R. T. (2017). Using connectome-based predictive modeling to predict individual behavior from brain connectivity. *Nature Protocols*, *12*(3), 506–518. https://doi.org/10.1038/nprot.2016.178

Shrout, P. E., & Fleiss, J. L. (1979). Intraclass Correlations: Uses in Assessing Rater Reliability. *Psychological Bulletin*, *86*(2), 420–428.

Varoquaux, G., Raamana, P. R., Engemann, D. A., Hoyos-Idrobo, A., Schwartz, Y., & Thirion, B. (2017). Assessing and tuning brain decoders: Cross-validation, caveats, and guidelines. *NeuroImage*, *145*(October 2016), 166–179. https://doi.org/10.1016/j.neuroimage.2016.10.038
